# Divergent growth and wood anatomical responses of European larch and Swiss stone pine to climate variability at alpine treeline ecotones

**DOI:** 10.3389/fpls.2026.1813904

**Published:** 2026-08-06

**Authors:** Francesco Marotta, Jan Tumajer, Krešimir Begović, Jernej Jevšenak, Hana Kuželová, Martin Lexa, Jiří Mašek, Nikolaus Obojes, Miloš Rydval, Václav Treml, Jelena Lange

**Affiliations:** 1 Department of Physical Geography and Geoecology, Faculty of Science, Charles University, Prague, Czechia; 2 Department of Forest Ecology, Czech University of Life Sciences Prague, Prague, Czechia; 3 Department for Forest and Landscape Planning and Monitoring, Slovenian Forestry Institute, Ljubljana, Slovenia; 4 Department of Wood Processing and Biomaterials, Czech University of Life Sciences Prague, Prague, Czechia; 5 Institute for Alpine Environment, Eurac Research, Bolzano, Italy; 6 Institute of Botany and Landscape Ecology, University of Greifswald, Greifswald, Germany

**Keywords:** climate change, climate-growth relationships, earlywood, *Larix decidua*, Pinus cembra, quantitative wood anatomy

## Abstract

Climate change is reshaping high-elevation forests in the European Alps, with species- and site-specific differences in the climate sensitivity of conifers being increasingly reported in treeline ecotones. However, a detailed understanding of how two dominant and co-occurring species, *Larix decidua* and *Pinus cembra*, respond to climate variability at inter- and intra-annual temporal scales and across treeline ecotones with different environmental settings is lacking. To address this gap, we analyzed chronologies of wood anatomical traits (earlywood lumen area, EWLA, and latewood cell wall thickness, LWCWT), tree-ring widths, and basal area increments (BAI) from five treeline ecotone sites across three Alpine regions with differing moisture regimes. We assessed climate-growth correlations, pointer years, long-term growth trends, and interspecific differences in absolute radial growth and wood anatomical traits. Our results show that, next to temperature, precipitation and atmospheric drought (vapor pressure deficit) are important drivers of growth variability. Pointer-year analyses clearly showed the differential response of the two species: growth and wood anatomy of *P. cembra* strongly depended on moisture, especially at drier sites, while *L. decidua* responded more consistently to temperature. Growth trajectories diverged between species: *L. decidua* showed consistently positive BAI, EWLA, and LWCWT trends, whereas *P. cembra* exhibited site- and trait-dependent positive, stable, or declining growth trends. Generalized additive mixed models indicated that for BAI, these differences could partly be attributed to species-specific climate effects, while effects on EWLA trends were mostly of structural origin for both species. LWCWT trends differed between species and sites despite broadly similar late-season climatic effects, pointing to additional species- and site-specific temporal dynamics. Notably, clear interspecific differences emerged in wood anatomical traits: although both species had a similar mean ring width, *L. decidua* consistently exhibited larger EWLA and LWCWT. Our findings highlight the contrasting mechanistic and ecological strategies of the two species, i.e., the isohydric, drought-sensitive, conservative *P. cembra* versus the anisohydric, efficiency-oriented and more temperature-limited *L. decidua*. Continued warming and intensifying drought may contribute to divergent growth trajectories between mature *L. decidua* and *P. cembra* especially in drier areas, with potential implications for future forest composition and growth dynamics in Alpine treeline ecotones.

## 1 Introduction

High-elevation mountain forests play a key role in regional biogeochemical cycles and ecological functioning, acting as important carbon sinks, regulating the water cycle, conserving biodiversity, stabilizing slopes, and providing ecosystem services that support local livelihoods (Körner, 2012). Yet changing climatic variability is reshaping these functions and raising management challenges of these ecosystems, with recent studies documenting emerging vulnerabilities in carbon storage capacity and other ecosystem services (Albrich et al., 2022; Hillebrand et al., 2023; Ioan et al., 2025). High-elevation forest ecosystems are especially vulnerable to climate change, as they occur near the physiological limits of tree survival and are highly sensitive to shifts in environmental conditions (Fritts, 1976). At alpine treeline, tree growth is primarily constrained by low temperatures, which limit cambial activity and the duration of wood formation (Körner, 2012). However, emerging water stress can counteract any positive effects of enhanced warming and extended growing season in cold environments by inhibiting tree hydraulic conductance (Tumajer et al., 2021; Obojes et al., 2022; Liang et al., 2014). Particularly, high vapor pressure deficit (VPD) has been associated with a shortened growing season in alpine treeline ecotones (Obojes et al., 2022). Climate warming in high-elevation forests may therefore differentially affect the distinct phases of wood formation depending on moisture conditions: warming may extend the growing season and accelerate wood formation (Rossi et al., 2007, 2016), yet higher temperatures may also amplify hydraulic pressure for tree growth, particularly in drier sites and during extended dry periods (Fajardo et al., 2019; Elliott et al., 2020; Liang et al., 2014). However, the interaction between the stimulating effects of increasing temperatures and the counteracting impacts of episodic droughts on intra-annual wood formation and radial growth remain poorly understood for trees in the alpine treeline ecotone.

In the European Alps, a pronounced hydroclimatic gradient, ranging from the wetter northern and southern edges to the drier inner-alpine basin, strongly influences forest species composition, adaptation to environmental changes, and productivity (Frei and Schär, 1998; Rigling et al., 2013). Ongoing climate change is altering these gradients by intensifying warming rates and shifting seasonal precipitation patterns, with climate projections indicating more frequent and severe droughts, affecting the already drier inner-alpine regions (Gobiet et al., 2013; Kotlarski et al., 2022). Two conifer species dominate the upper forest limits in the Central Alps: *Larix decidua*, a fast-growing deciduous pioneer, and *Pinus cembra*, a slow-growing evergreen. *L. decidua* often colonizes disturbed or bare soils and exhibits a more anisohydric behavior under moisture stress by keeping its stomata open longer under higher VPD, while *P. cembra* as a more isohydric species, closes stomata early during hydraulic stress (Anfodillo et al., 1998; Wieser, 2012). Previous dendrochronological studies in the Alps have shown that *P. cembra* growth is constrained by low atmospheric humidity and high VPD, especially in dry sites (Obojes et al., 2022), while *L. decidua* is generally less sensitive to such water-related stress (Tumajer et al., 2025). For both species, a weakening of summer temperature-growth relationships has recently been observed in high mountains such as the Tatra Mountains (Izworska et al., 2025), the south-western Carpathians (e.g., Știrbu et al., 2022), and the Alps (Leonelli et al., 2009). Despite extensive dendrochronological research on these species in treeline ecotones in the Alps (Carrer et al., 2007; Frank and Esper, 2005; Büntgen et al., 2007; Obojes et al., 2022, 2024), most of the existing knowledge is based on annually resolved tree-ring width (TRW) analyses that may offer limited information on the intra-annual responses of tree growth to climate (but see Carrer et al., 2018; Unterholzner et al., 2024).

Quantitative wood anatomy (QWA) can provide a more detailed and mechanistic insight into tree growth responses to climate through analyzing xylem anatomical traits such as lumen area and cell wall thickness at intra-annual resolution (Piermattei et al., 2025). These traits form during distinct phases of cambial activity throughout the growing season and thus respond to different climatic drivers (Cuny et al., 2014). By separately assessing these phases, QWA can reveal seasonal climate influences often masked in annually resolved TRW series. Although wood anatomical traits can be influenced by tree size and allometric constraints (Kašpar et al., 2019), earlywood traits tend to also be sensitive to weather conditions during the late spring-early summer cell enlargement phase (Gruber et al., 2009; Fonti et al., 2010), whereas latewood traits are influenced by temperature during late-season cell wall deposition and lignification (Fonti et al., 2010; Cuny and Rathgeber, 2016). Because earlywood tracheid enlargement is largely driven by cell turgor, drought-induced reductions in plant water status can constrain lumen expansion and lead to smaller earlywood lumen area (Eilmann et al., 2011; Știrbu et al., 2022). Although lumen area, latewood tracheid number and wall thickness in *P. cembra* at treeline have been shown to respond positively to summer warmth (Carrer et al., 2018; Unterholzner et al., 2024), lumen area is more strongly constrained by water availability, decreasing under drought-related or high-evaporative-demand conditions that limit tracheid enlargement (Știrbu et al., 2022; Unterholzner et al., 2024) and suggesting potential constraints on hydraulic capacity under drier conditions. For *L. decidua*, both positive effects of temperature (Carrer et al., 2017; Cuny et al., 2018; Rozenberg et al., 2020) and negative effects of drought (Rozenberg et al., 2020) on radial growth and wood formation have been reported as well. In terms of absolute growth, it was found that *L. decidua* typically forms larger earlywood tracheids and exhibits thicker latewood cell walls than *P. cembra* when growing under the same conditions (Rossi et al., 2009; Cartenì et al., 2018), consistent with a more efficiency-oriented xylem structure. Collectively, these studies highlight that investigating species- and trait-specific growth responses using approaches that go beyond annually derived tree growth metrics can provide precise insights into tree growth dynamics under climate change. Despite the growing body of literature on this topic, to our knowledge, no study has jointly examined co-occurring *P. cembra* and *L. decidua* across multiple Alpine treeline ecotones spanning a gradient of moisture availability while simultaneously integrating climate–growth relationships, pointer-year responses, long-term growth trends, and absolute growth derived from both annual and intra-annual growth records. Such an integrative framework could provide a more mechanistic understanding of species- and site-specific responses to changing climatic conditions, including climatic extremes, and help identify the seasonal conditions and potential climatic thresholds associated with exceptionally high or low growth. Such empirical benchmarks are critical for improving predictions of species-specific growth responses under ongoing climate change.

In this study, we assessed climate-growth responses of *L. decidua* and *P. cembra* at three alpine treeline sites spanning a moisture gradient: a humid site at the northern edge of the Alps (Karwendel Valley, Austria) and two drier inner-alpine sites (Mazia Valley, Italy; S-charl Valley, Switzerland). We combined the analysis of two traditional tree-ring metrics, TRW and basal area increment (BAI) with QWA, deriving earlywood lumen area (EWLA) and latewood cell wall thickness (LWCWT) chronologies for both species. We studied three treeline sites, including two with opposing slopes, resulting in 20 wood anatomical chronologies from five sites over the period 1980-2022. We then conducted (i) partial bootstrap correlations between growth variables and seasonal climatic means calculated from daily data; (ii) a pointer-year analysis for TRW and EWLA to link growth deviations with climatic anomalies and climatic envelopes; and (iii) assessed long-term growth trends, their drivers, and absolute growth in BAI/TRW, EWLA and LWCWT.

We tested the following hypotheses:

1. In addition to (prevailing) positive correlations of TRW and LWCWT with summer temperature, TRW and EWLA are positively correlated with spring-summer precipitation and negatively correlated with spring-summer VPD, with stronger relationships for *P. cembra* and at the drier sites.
2. Growth responses during climatically extreme years are species-specific, with prevailing positive responses of *P. cembra* TRW and EWLA to wetter conditions, and with prevailing positive responses of *L. decidua* TRW and EWLA to warmer conditions.
3. The generally higher drought sensitivity of *P. cembra* compared to *L. decidua* is reflected in comparatively lower growth rates and in lower absolute radial growth and reduced anatomical traits (EWLA and LWCWT) relative to *L. decidua*.

## 2 Material and methods

### 2.1 Study sites

**Figure 1:**
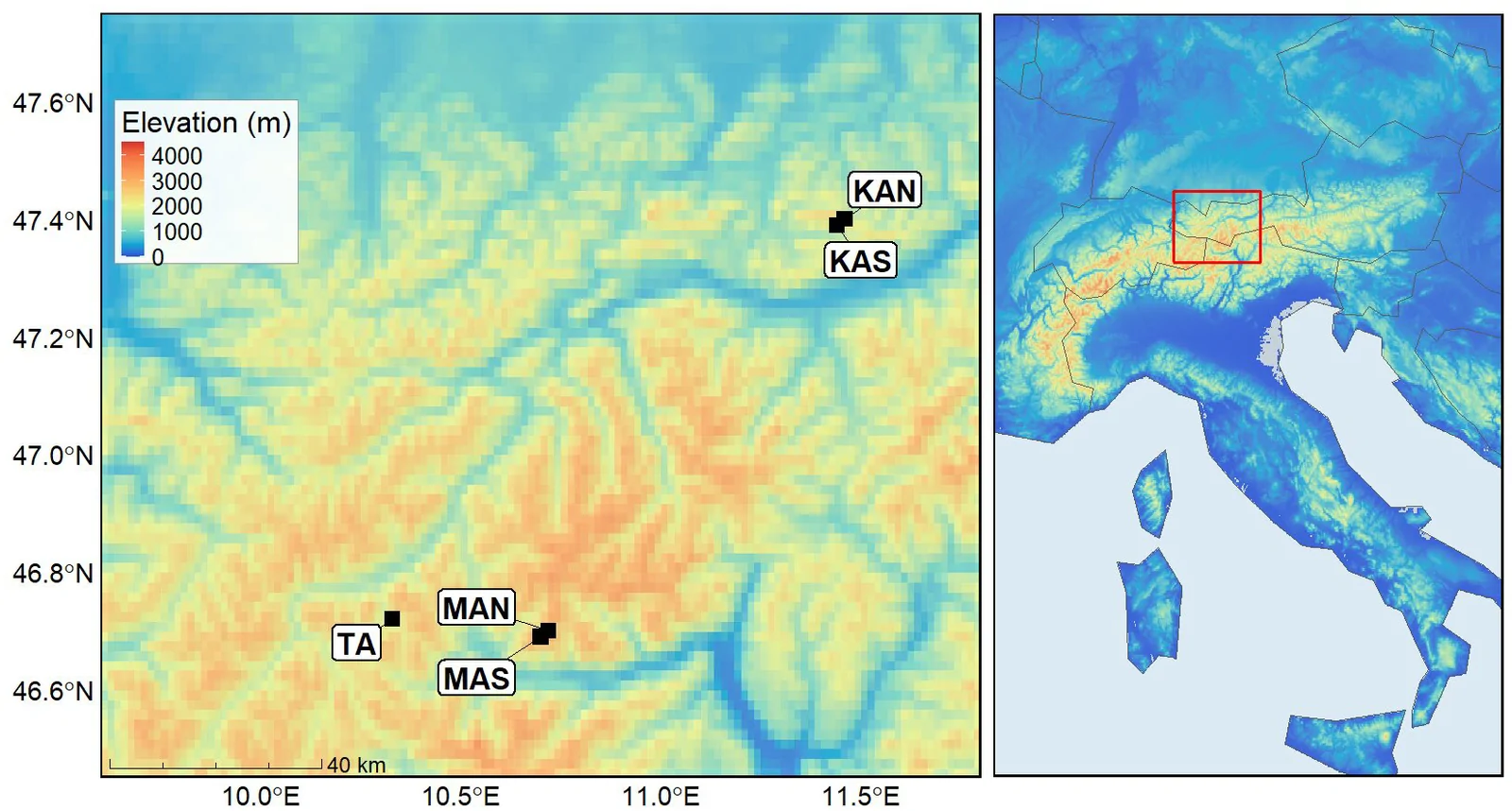
Geographical location of the study sites in the European Alps. Site abbreviations indicate region and slope aspect: MAN and MAS refer to the north- and south-facing slopes in Mazia Valley, Italy; KAN and KAS refer to the north- and south-facing slopes in Karwendel, Austria; and TA refers to the S-charl site, Switzerland. The map was created with QGIS 3.42.2.

The study was carried out in three treeline ecotones in the Central-Eastern Alps across Italy, Austria, and Switzerland. The sites are located in the Mazia Valley (MA), South Tyrol, Italy; Karwendel Mountains (KA), Tyrol, Austria; and S-charl Valley (TA), Graubünden, Switzerland (**Figure 1**). To account for contrasting slope exposures, north- and south-facing slopes were selected at Mazia (MAN, MAS) and Karwendel (KAN, KAS), whereas TA was northeast-facing, resulting in a total of five study sites.

The Mazia Valley is a side valley of the Vinschgau/Venosta Valley in western South Tyrol and is characterized by a dry inner-alpine continental climate (Obojes et al., 2024). It is among the driest regions of the European Alps, with a mean annual temperature of 6.4 °C and mean annual precipitation of 682 mm at the nearby Monte Maria climate station (1310 m a.s.l.). The forest line is located at approximately 2250 m a.s.l. on south-facing slopes and 2320 m on north-facing slopes.

Val S-charl, located in the Lower Engadine region (canton of Graubünden, Switzerland), exhibits a similarly continental climate. At 1970 m a.s.l., mean annual temperature is 0.94 °C and annual precipitation averages 871 mm (MeteoSwiss station Buffalora). The uppermost *Pinus cembra* trees currently occur at around 2500 m a.s.l., well above the prominent forest edge near 2300 m a.s.l., which reflects historical land use rather than the climatic treeline (Körner and Hiltbrunner, 2024).

The Karwendel Mountains, situated in the Northern Limestone Alps north of Innsbruck (Tyrol, Austria), experience a humid alpine climate. Mean annual precipitation ranges from 1500 to 1750 mm and mean annual temperature is approximately 4 °C at 1500 m a.s.l. The upper treeline occurs between ~1800 and 1900 m a.s.l., depending on local topography and microclimate (Schiechtl et al., 1987).

### 2.2 Sample collection and processing

Fieldwork was carried out during two sampling campaigns in 2022 (MA, KA) and 2023 (TA). At the current upper tree limit, a total of 205 trees (18 to 26 individuals per site and species, see **Supplementary Table 1** for site details) were sampled from mixed subalpine stands composed of *P. cembra* and *L. decidua*, selecting mature dominant trees with no signs of mechanical injury or disease. Two cores per tree were taken with an increment borer (Haglöf, 5.15 mm in diameter) parallel to the slope to minimize the occurrence of reaction wood and possible bias due to asymmetric growth. Dendrometric parameters for each sampled tree were recorded to evaluate tree morphology and stand structure at the site level, including tree height and diameter at breast height (DBH). Sampled cores were air-dried and fixed on wooden laths using a water-soluble mounting glue. Wood surfaces were sanded with progressively finer grit sandpaper or prepared with a sledge microtome (Gärtner and Nievergelt, 2010) to enhance ring boundary visibility. The samples were then scanned using a digital high-resolution scanner (1200–2400 dpi), and tree-ring widths (TRW) were measured using the CooRecorder software with nominal 0.001 mm resolution (v. 9.8.1; Maxwell and Larsson, 2021). Visual and statistical dating was applied to each series (Holmes, 1983) using the CDendro software (v. 9.8.1, Maxwell and Larsson, 2021). To remove long- and medium-term biological growth trends and retain high-frequency growth variability, each TRW series was detrended by fitting a 30-year cubic smoothing spline with a 50% frequency cut-off (Cook et al., 1990; Klesse and Bigler, 2025). The detrended series were averaged with a robust mean to generate 10 site- and species-specific standard chronologies corresponding to the five study sites and two species, using the R package dplR (v. 1.7.8) (Bunn, 2008; R Development Core Team, 2018).

Based on the raw TRW measurements, basal area increments (BAI) were computed for each tree. Since DBH data was available, we used the outside-in method (bai.out function from the dplR package) as recommended by Klesse and Bigler (2025). This geometric approach converts multiple ring-width series into BAI from the bark towards the pith, considering DBH measurements. Same as TRW, BAI series were detrended by fitting a 30-year window cubic smoothing spline with a 50% frequency cut-off (Cook et al., 1990). Site- and species-specific standard chronologies were built from detrended (climate-growth correlations) and raw (trend analysis) BAI using the robust mean. All TRW and BAI chronologies were cropped to 1950–2022 for subsequent analyses.

For the wood anatomical processing and measurement, we selected a subsample of five trees per site and species, resulting in a total of 50 trees. To minimize potential age- and size-related effects (Carrer et al., 2015), only trees of mature age (i.e., > 150 rings) and of similar height were included in the analysis. Selected trees were chosen based on sample quality and representativeness, excluding individuals with visible defects such as cracks, decay, or compression wood. To verify that the subset correlated well with the respective site chronology, we calculated Pearson correlations between the TRW series of each selected tree and the corresponding site- and species-specific master TRW chronology (**Supplementary Table 2**). The selected samples were removed from wooden mounts and divided into segments of about 4 cm length, which were then dehydrated using the tissue processor (Leica ASP200) and embedded in paraffin using an embedding station (Leica HistoCore Arcadia H) to stabilize cells and facilitate a smoother and more precise sectioning. Transversal micro-sections of 10-12 µm thickness were then obtained from each segment using a rotary microtome (Leica RM 2125 RTS), following standard protocols for wood anatomical preparation and sectioning (von Arx et al., 2016; Fonti et al., 2025). The micro-sections were stained with a 1% safranin and a 0.5% of astrablue solution, washed and dehydrated through an ethanol series (50% to 95%), and prepared as permanent slides using Euparal. The micro-sections were scanned at 10x magnification using a slide scanning system (ZEISS Axioscan Z.1), which captures entire sections at high resolution under standardized settings. Anatomical parameters such as cell lumen area (LA) and radial and tangential cell wall thickness (CWT) were measured from the scanned images using the semi-automated software Roxas v. 3.0.655 (von Arx and Carrer, 2014; Prendin et al., 2017) for the period 1980-2022. The dating of the wood anatomical series was verified using the Roxas and the R software. Through R-based (v 4.5.0) processing (R Development Core Team, 2018), each tree ring was divided into earlywood (EW) and latewood (LW) applying a Mork index of 1 (Denne, 1988) to capture functional differences at the intra-annual scale (Cuny et al., 2014; Cartenì et al., 2018). For each EW and LW part, the 90^th^ percentile of LA (Castagneri et al., 2017) and the mean of radial and tangential CWT (Prendin et al., 2017) were calculated. We focused on LA of the EW (EWLA) and CWT of the LW (LWCWT) as ecologically meaningful representatives of intra-annual growth processes, with EWLA reflecting early-season hydraulic capacity and LWCWT reflecting late-season investment in cell wall thickening (Fonti et al., 2010). Same as TRW and BAI, each wood anatomical series was standardized by fitting a 30-year cubic smoothing spline with a 50% frequency cut-off following (Cook et al., 1990). The detrended time series of EWLA and LWCWT of each tree were averaged using the robust mean to produce site- and species-level standard chronologies for each variable and tree-ring sector, resulting in a total of 20 wood anatomical chronologies (5 sites x 2 tree-ring sectors x 2 species; 1980-2022). Chronology quality was assessed using standard statistics (mean inter-series correlation, rbar; and expressed population signal, EPS) calculated for all site × species chronologies, including TRW (**Supplementary Table 1**) and the anatomical series (EWLA and LWCWT; **Supplementary Table 3**) to evaluate the strength of the common signal.

### 2.3 Climatic data

We obtained daily mean, minimum, and maximum temperature, total precipitation, and relative humidity derived from the gridded E-OBS dataset (v. 30.0) at 0.1° spatial resolution for the period 1950-2022 (Cornes et al., 2018). To account for topographic effects in heterogeneous alpine environments, climate data were elevation-corrected separately for each site using local geostatistical interpolation (Goovaerts, 2000; Adhikary et al., 2017; Jevšenak et al., 2021). For each site, all E-OBS grid cells within a ±0.5° latitude/longitude window around the sampling location were extracted, together with their corresponding E-OBS grid-cell elevation. Climate values were then predicted at the exact site coordinates and site elevation using universal kriging with elevation as an external drift, implemented in R with the sf (v. 1.1), gstat (v. 2.1), and automap (v. 1.1) packages (Pebesma, 2004; Hiemstra et al., 2009; Pebesma, 2018). For each day and climate variable, the local climate field was modelled as a function of elevation and spatially autocorrelated residual variation, with empirical variograms fitted automatically before site-level prediction. Temperature and relative humidity were interpolated on their original scale, while precipitation was log-transformed before interpolation and back-transformed to the original scale to reduce skewness and avoid negative predictions. Interpolation uncertainty was assessed using kriging prediction variance and leave-one-grid-cell-out cross-validation within each local extraction window. Finally, climate data were detrended same as TRW, BAI, and wood anatomical chronologies using a 30-year cubic smoothing spline with a 50% frequency cut-off to allow climate variables and growth proxies to be compared within a similar frequency domain (Ols et al., 2023). To evaluate the sensitivity of this methodological choice, we repeated the analyses using non-detrended climate data. The resulting patterns were highly similar to those obtained with the 30-year spline detrended climate series (**Supplementary Figure S1**). Additional sensitivity tests using 10- and 70-year splines for both growth and climate series detrending showed slight differences in the magnitude and range shifts of a few correlations, but did not alter the main ecological conclusions obtained based on the 30-year spline standardized chronologies (**Supplementary Figures S2**, **S3**). VPD was derived from relative humidity and air temperature using the RHtoVPD function in the R package plantecophy*s* (v. 1.4-6), which converts relative humidity (%) to VPD (kPa) based on temperature-derived saturation vapor pressure (Duursma, 2015). Because VPD is derived from air temperature and relative humidity, we assessed collinearity among the main climatic variables. Variance inflation factors (VIFs) were calculated for the March-August period (**Supplementary Table 4**), representing the main period covered by most of the detected climate-growth relationships. Collinearity was low to moderate among the variables used in the analyses (mean temperature, precipitation, and VPD; VIF 2.55-3.16; **Supplementary Table 4**), but high when relative humidity was included (VIF 6.99-7.43). This indicates that collinearity did not preclude the joint inclusion of temperature, precipitation and VPD in the same analytical framework, although temperature- and VPD-related effects should be interpreted cautiously because VPD partly reflects temperature-driven atmospheric demand.

Temperature and precipitation data for 1980–2022 from the respective nearest meteorological stations (Monte Maria for MA, ~1300 m; Seefeld for KA, ~1200 m; and Buffalora for TA, ~2000 m, **Supplementary Figure S4**-**S6**) showed the following trends: Mean annual temperatures increased significantly at all sites, with total rises of +2.9 °C (MA), +1.5 °C (KA), and +1.2 °C (TA). No significant long-term trends in annual precipitation sums were detected. However, precipitation at TA showed a significant decrease in spring (March-May), and the spring precipitation decline was almost statistically significant at MA.

### 2.4 Statistical analyses

#### 2.4.1 Static climate-growth relationships

Climate-growth correlations were calculated between detrended TRW, BAI, EWLA, and LWCWT chronologies and daily climate over the period 1980-2022. To disentangle the individual effects of temperature, precipitation, and VPD on tree growth, we calculated partial bootstrap correlations using the dendroTools package in R (v. 1.2.15) (Jevšenak and Levanič, 2018; Jevšenak, 2020). To obtain robust estimates of correlation coefficients and their significance, the bootstrap was calculated using 200 iterations with normal-based 95% confidence intervals (α = 0.05). Average values of mean, maximum, and minimum temperatures, total precipitation and VPD were calculated for consecutive time windows in steps of one day and ranging from 14 to 60 days in length, from January 1 until September 30. For each combination of window length and position of its center, we calculated the linear correlation between the window-averaged climate variable and the growth series, generating a detailed correlation matrix for each site and species. Notably, these seasonal windows were not restricted to calendar months but could include any possible period within the defined range. For each climate-growth correlation, we accounted for potential confounding climatic factors by including a relevant control variable in the partial correlations: we assessed the relationship between growth and precipitation while controlling for temperature, between growth and temperature while controlling for precipitation, and between growth and VPD while controlling for temperature.

Because daily-resolved climate-growth analyses involve a large number of overlapping temporal windows, which may inflate correlation coefficients and increase the risk of spurious significance (Torbenson et al., 2025), we focused our interpretation on coherent and biologically plausible correlation patterns that were consistent across sites and/or species, rather than on isolated significant correlations.

#### 2.4.2 Pointer years in tree-ring width and wood anatomical traits

We analyzed whether extreme climatic conditions coincided with exceptionally wide and narrow TRW and EWLA over the period 1980-2022. These two growth proxies were selected because TRW reflects overall annual growth, while EWLA captures growth responses to early-season climate (Fonti et al., 2010; Cuny et al., 2014). We identified years with growth anomalies as those in which detrended TRW or EWLA values were at least 1.5 times the standard deviation above or below the mean of the respective chronology per site and species (Belokopytova et al., 2022; Frank et al., 2005). This approach allows to detect years with exceptionally high or low growth, potentially linked to anomalous climatic conditions (Jetschke et al., 2019). We then assessed climate anomalies during the pointer year and in the preceding and following year to evaluate whether exceptionally large or small TRW and EWLA were associated with coherent climatic conditions before, during or after the growth anomaly.

To do so, we selected predefined seasonal climate windows with presumably large influence on the formation of each proxy: May-July for TRW, corresponding to the main period of radial growth, and May-June for EWLA, corresponding to the main period of earlywood formation (Rossi et al., 2007; Gruber et al., 2009; Cuny et al., 2014). For each proxy and site × species chronology, we calculated mean temperature, total precipitation and mean VPD over the corresponding fixed seasonal window from daily climate data for the pointer years. Furthermore, we calculated empirical climatic envelopes for pointer years, defined here as the central range of observed seasonal temperature, precipitation and VPD conditions associated with exceptionally high or low TRW or EWLA. For each pointer-year type, growth proxy, species and site, these envelopes were summarized using the interquartile range (Q25–Q75).

We also assessed whether pointer years were synchronized among growth proxies, sites and species. Each site × species × year event was assigned to one of the three categories: TRW-only, EWLA-only, or shared TRW and EWLA. Synchrony was evaluated by counting the recurrence of the same calendar years across chronologies, both within species and between species, and separately for TRW and EWLA.

#### 2.4.3 Long term growth trends and absolute growth

To analyze long-term growth dynamics, we examined the growth trends of BAI over the period 1950–2022 and of EWLA and LWCWT over the period 1980–2022 separately for each site and species. These growth proxies were selected since they represent complementary information of tree growth. Tree age distribution was inhomogeneous across sites and species, partly including relatively young trees and with significant differences among sites for both species (*P. cembra*: χ² = 17.09, df = 4, p = 0.0019; *L. decidua*: χ² = 18.76, df = 4, p < 0.001; **Supplementary Figure 7**, **Supplementary Table 5**). Due to this setting, a subsample was created for the trend analysis with trees that feature 150 to 300 rings in 2022/2023 to ensure a comparable age structure and minimize possible age-related trends, especially in BAI, (Klesse and Bigler, 2025) and EWLA (Carrer et al., 2015). This age filtering reduced the sample size to 11 individuals at MAS, 8 at MAN, 12 at KAN, 16 at KAS, and 16 at TA for *P. cembra*, and to 15 at MAS, 11 at MAN, 18 at KAN, 19 at KAS, and 21 at TA for *L. decidua*. Trends were represented graphically and interpreted using the non-detrended series to maintain long-term variability and highlight any directional changes in growth.

To disentangle climatic, ontogenetic, and residual temporal components contributing to the long-term growth trajectories, we fitted tree-level generalized additive mixed models (GAMMs) separately for BAI, EWLA and LWCWT using the R package mgcv (v. 1.9; Wood, 2017). Climatic predictors were selected based on the biological meaning and main formation period of each proxy, while also considering the dominant climate-growth signals detected in the correlation analyses. Cambial age, reconstructed stem diameter (an annually resolved time series calculated from the measured DBH and annual growth increments), and tree height were evaluated as predictors of the ontogenetic component. For each growth proxy, candidate models were compared using the Akaike Information Criterion, ΔAIC (Akaike, 1974), deviance explained, and adjusted R² to evaluate whether additional structural or climatic terms improved model performance. For BAI, cambial age and reconstructed stem diameter were retained in the models as ontogenetic predictors, and May–July temperature, precipitation and VPD were included as climatic predictors (**Supplementary Table 6**, **S8**). Given the additional winter-spring VPD sensitivity detected for *P. cembra*, December-February and March-May VPD terms were also included in the Pinus-specific model. For EWLA, cambial age, reconstructed stem diameter and tree height were included to account for ontogenetic development, and May–June temperature, precipitation and VPD were included as climate predictors. For LWCWT, we retained cambial age and reconstructed stem diameter, together with July–August temperature, precipitation and species-specific VPD effects, corresponding to the main period of latewood cell wall thickening and lignification.

All continuous predictors were standardized before model fitting. Collinearity among ontogenetic and climatic predictors was assessed using pairwise correlations and VIF/GVIF diagnostics, and concurvity was evaluated for the GAMM smooth terms (**Supplementary Tables 9**, **10**). All models included site × species as a fixed effect, tree identity as a random effect, and an AR(1) structure to account for temporal autocorrelation of the growth series. In addition, site × species-specific smooths of calendar year were included to test whether residual temporal structure remained after accounting for structural and climatic predictors. Full model specifications, selection results, and smooth-term summaries are reported in **Supplementary Tables 6**–**16**.

Using the same age-filtered dataset and non-detrended growth series, we further assessed between-species differences in absolute radial growth and wood anatomical traits. Annually resolved site-level chronologies were analyzed using linear mixed-effects models, using the R package lme4 (v. 1.1; Bates et al., 2015), to account for the hierarchical structure of the data and temporal autocorrelation. In the final model, the annual site-level chronology value was used as the response variable, while species, growth proxy identity (TRW, EWLA and LWCWT), their interaction, and region were included as fixed effects, and site was included as a random effect nested within site pair. Temporal autocorrelation was modelled using a continuous AR(1) correlation structure within each site-level proxy chronology, and proxy-specific residual variances were allowed to differ among TRW, EWLA and LWCWT (**Supplementary Table 17**). Between-species differences for each proxy were evaluated using estimated marginal means and pairwise contrasts, with Holm adjustment (Holm, 1979) for multiple comparisons (**Supplementary Tables 18**, **19**). As a sensitivity analysis, we fitted an additional model including slope aspect as a fixed effect after excluding S-charl, where slope aspect was not available (**Supplementary Table 20**). Results were visualized as boxplots showing the distribution of annual site-level values by species.

## 3 Results

### 3.1 Climate-growth correlations

**Figure 2:**
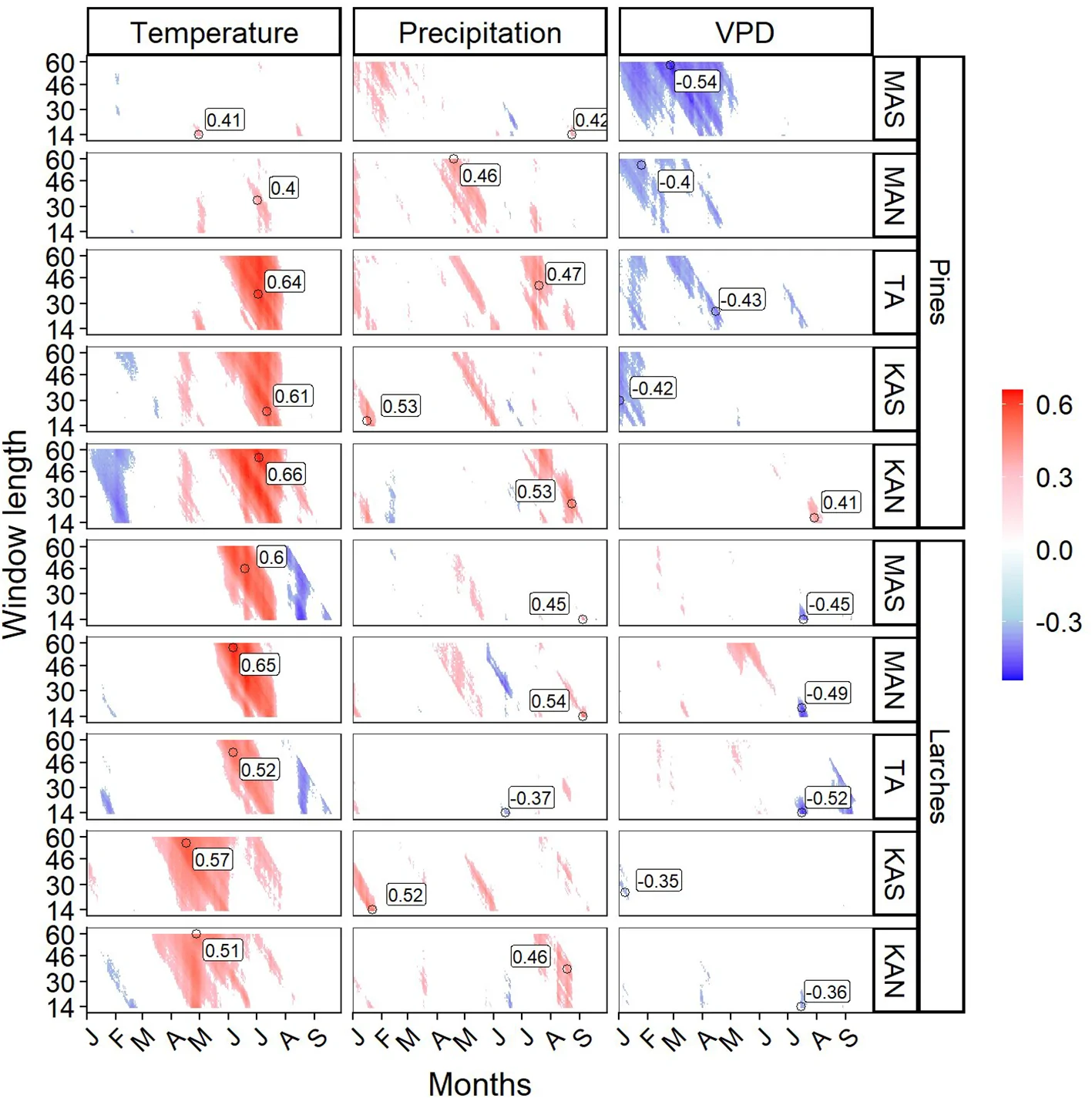
Partial bootstrap correlations between seasonal climatic means derived from daily climate E-OBS data (mean temperature, total precipitation, and VPD) and tree-ring width chronologies by site and species. Letters (x axes) indicate the months of the current year from January to September. The length of the considered seasons ranges from 14 to 60 days (y axes). The respective correlations are centered over the respective day of year (x axes) and duration (y axes). Only significant correlations are shown (p<0.05). The strongest significant correlation of each subplot is indicated by a circle and the respective value.

Across most sites and for both species, TRW showed consistently strong positive correlations with summer temperature (**Figure 2**), except for *P. cembra* at the dry Mazia sites (MAS, MAN), where positive temperature responses were weaker and restricted to narrower temporal windows. Notably, using mean, minimum, or maximum temperatures yielded comparably uniform correlations, indicating that the observed patterns were not dependent on the specific temperature metric used (**Supplementary Figure 8**). VPD responses differed between species: *P. cembra* TRW showed a pronounced negative response to VPD during winter-spring at the relatively drier MAN, MAS, TA and KAS sites. For *L. decidua*, a short period of negative correlation emerged in relation to late summer VPD at most of the sites. In line with this, *P. cembra* showed a clearer positive response to spring-summer precipitation compared to *L. decidua*. Similar patterns were observed for BAI, which closely mirrored the correlations found for TRW (**Supplementary Figure 9**).

**Figure 3:**
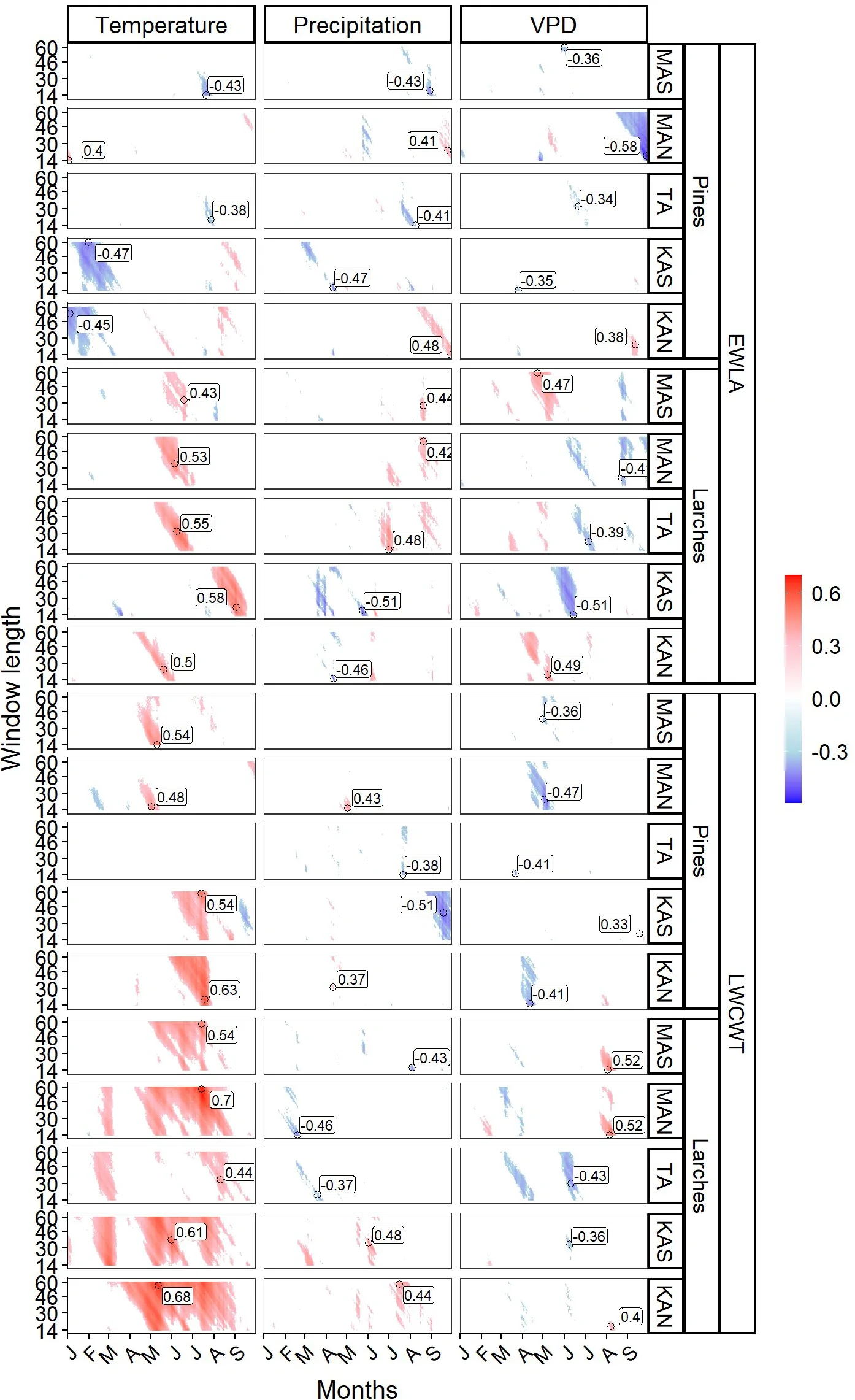
Partial bootstrap correlations between seasonal climatic means derived from daily climate E-OBS data (temperature, total precipitation, and VPD) and earlywood lumen area (EWLA) and latewood cell wall thickness (LWCWT) by site and species. Letters (x axes) indicate the months of the current year from January to September. The length of considered seasons ranges from 14 to 60 days (y axes). The respective correlations are centered over the respective day of year (x axes) and duration (y axes). Only significant correlations are shown (p<0.05). The strongest significant correlation of each subplot is indicated by a circle and the respective value.

Correlation patterns of anatomical traits were consistent with those of TRW but revealed additional seasonal details (**Figure 3**). LWCWT showed pronounced positive correlations with spring to summer temperatures at most sites and for both species (**Figure 3**), with considerably stronger correlations for *L. decidua* and at the colder/wetter KAN, KAS, and MAN sites. EWLA-temperature responses were species-specific: EWLA of *P. cembra* showed a pronounced negative response to winter temperatures at KAN and KAS. In contrast, EWLA of *L. decidua* responded positively to growing season temperatures at most sites. Correlations were largely consistent regardless of whether mean, minimum, or maximum temperatures were used (**Supplementary Figure 10**). EWLA response patterns related to precipitation and VPD were weaker and less clear.

### 3.2 Pointer years in tree-ring width and wood anatomical traits

Analyses related to negative pointer-years did not reveal consistent patterns across proxies, species or sites. Therefore, the main text focuses on positive pointer years, whereas the corresponding negative pointer-year results are reported in the **Supplementary Material** (**Supplementary Figure S11**; **Supplementary Table 21**).

**Figure 4:**
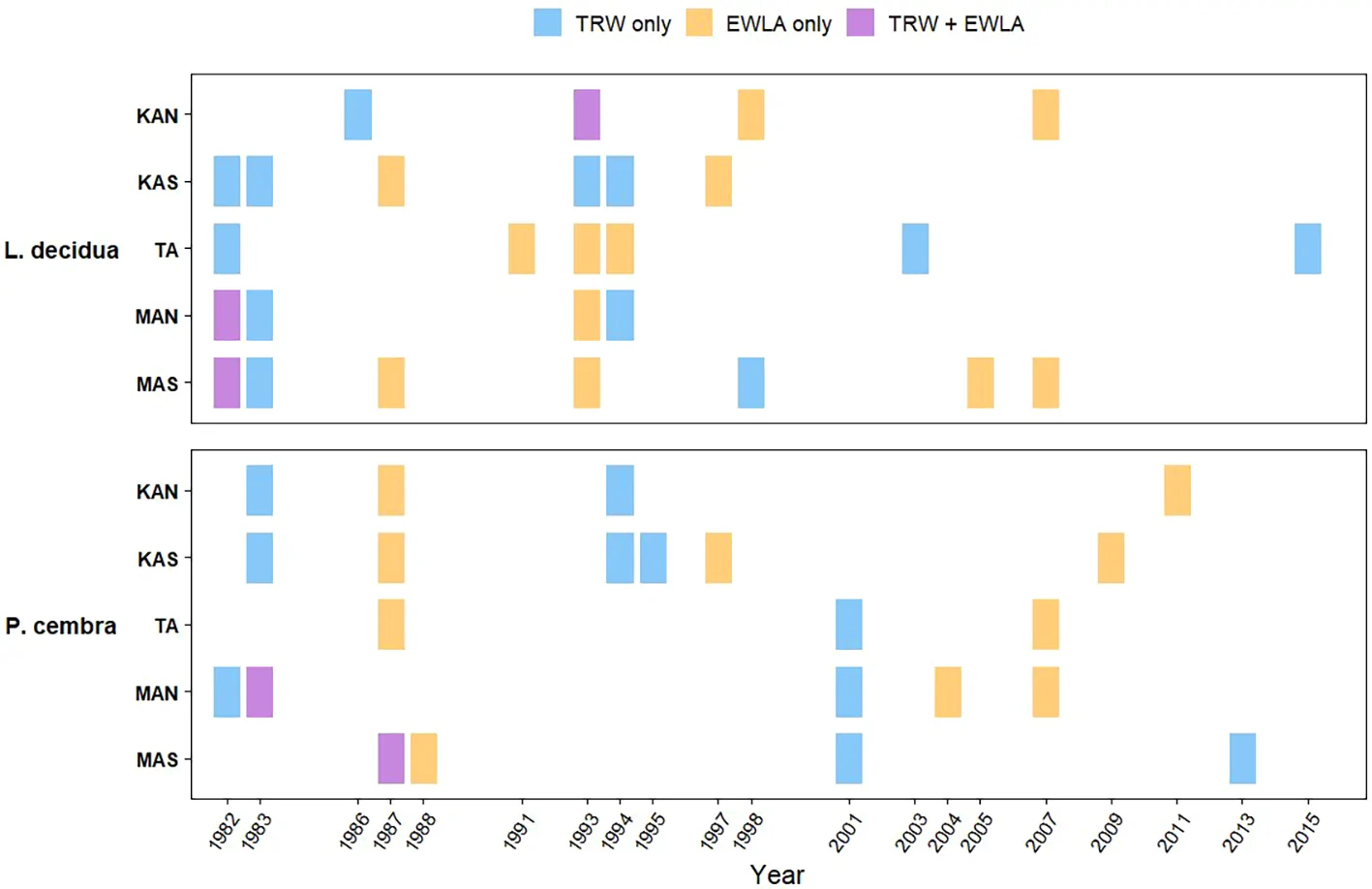
Tile plot showing positive pointer years detected in tree-ring width (TRW) and earlywood lumen area (EWLA) per species and site. Colors indicate whether a given year was identified as a positive pointer year for TRW only (blue), EWLA only (yellow), or for both proxies simultaneously (purple). Positive pointer years were defined as years in which the respective chronology exceeded the mean by at least 1.5 standard deviations.

Synchrony of positive pointer years of both growth proxies was highest within species, lower between species, and rare between TRW and EWLA (5 shared events; **Figure 4**). For TRW, notable pointer years that were shared by both species and several sites occurred in 1982, 1983 and 1994. For EWLA, common positive pointer years occurred in 1987, 1997 and 2007.

**Figure 5:**
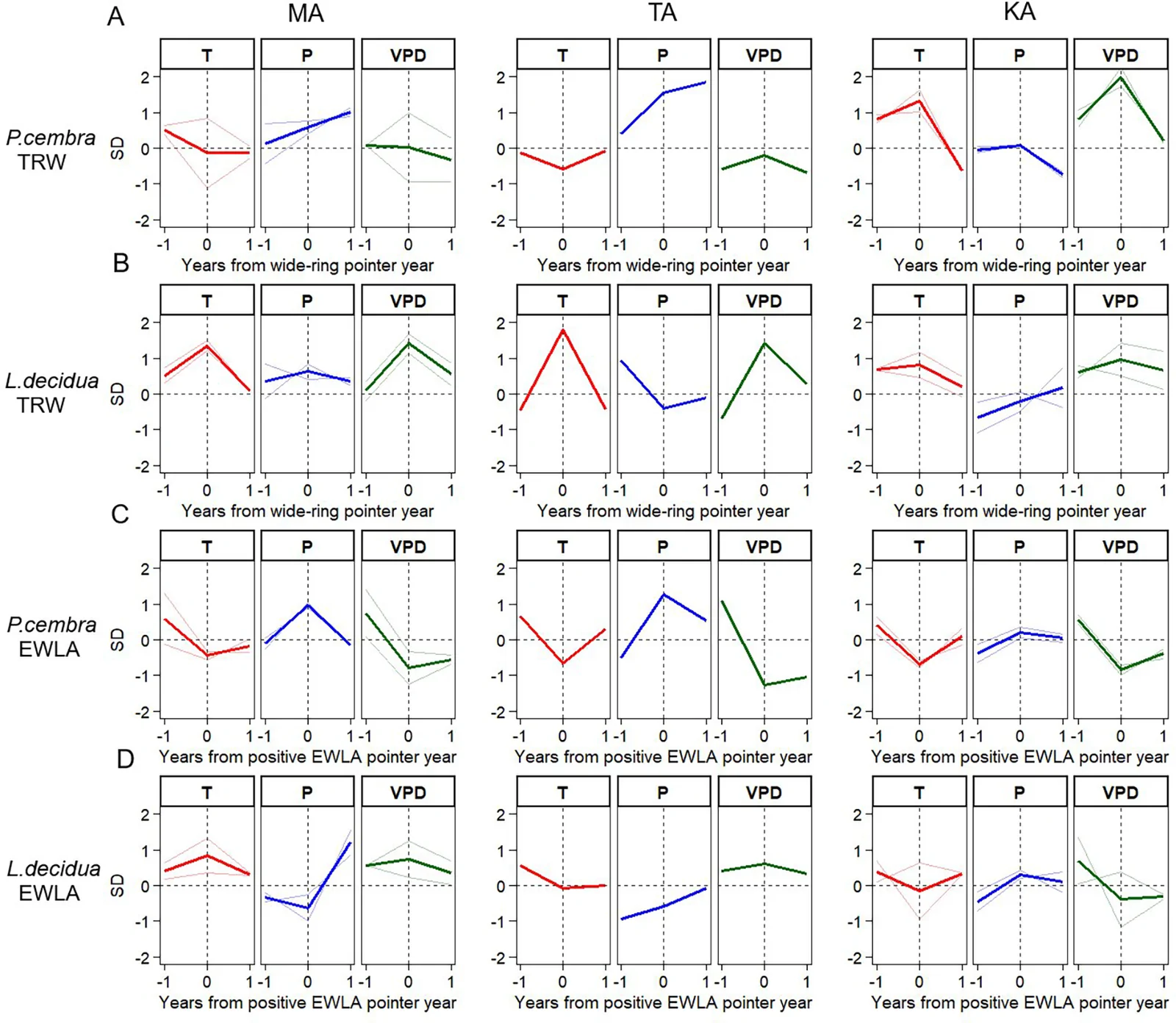
Climate anomalies associated with exceptionally wide tree rings (TRW) and large earlywood lumen area (EWLA) during positive pointer years. Positive pointer years were identified from standardized TRW chronologies [panels **(A, B)**] and EWLA chronologies **(C, D)** for *P. cembra* **(A, C)** and *L. decidua* **(B, D)**, using a threshold of +1.5 SD above the long-term mean. Standardized anomalies of mean temperature (T), precipitation sums (P), and vapor pressure deficit (VPD) are shown from one year before (-1) to one year after (+1) the pointer year (0). Climate variables were calculated over fixed seasonal windows: May–July for TRW and May–June for EWLA. Columns represent the three study regions: Mazia (MA), S-charl (TA), and Karwendel (KA). Thin lines indicate site-level climate anomalies, while thick lines represent the regional mean response. Horizontal dashed lines indicate zero climate anomaly, and vertical dashed lines mark the positive growth pointer year.

Pointer year analysis (**Figure 5**) showed species-specific results: exceptionally wide TRW in *L. decidua* was clearly associated with warmer-than-average and high-VPD conditions across all sites. In contrast, *P. cembra* showed this pattern only at the TA site, while wide rings were linked to wetter-than-average conditions at the drier MA and TA sites.

Positive pointer years in EWLA in *P. cembra* were consistently associated with below-average temperatures and VPD across sites and above-average precipitation at the drier MA and TA sites. Associations of *L. decidua* EWLA with climate were less clear, but wider EWLA was often linked with above-average temperature and VPD, and below-average precipitation. Patterns remained largely consistent with minimum and maximum temperature metrics, with only minor site- or species-specific differences observed (**Supplementary Figure 12**). The interquartile climatic envelopes associated with positive pointer years showed site-, species- and proxy-specific patterns (**Table 1**). For TRW, climatic conditions during a positive pointer year were generally colder at the drier/colder MAS–MAN–TA sites compared to the wetter KAS–KAN sites. For both TRW and EWLA, climatic conditions were generally colder and wetter for *P. cembra*, and warmer and drier for *L. decidua*.

**Table 1:** Empirical climatic envelopes for temperature, precipitation and vapor pressure deficit (VPD) calculated for positive pointer years of tree-ring width (TRW) and earlywood lumen area (EWLA) for each site and species over the period 1980–2022.

Site	Species	n	Temperature Q25-Q75 (°C)	Precipitation Q25-Q75 (mm)	VPD Q25-Q75 (kPa)
TRW (MJJ)					
MAS	*L. decidua*	3	8.8-9.38	302.03-351.59	0.33-0.37
MAS	*P. cembra*	3	6.89-7.17	308.56-361.75	0.26-0.29
MAN	*L. decidua*	3	9.15-9.38	335.92-373.28	0.36-0.39
MAN	*P. cembra*	3	8.34-9.38	330.99-368.35	0.32-0.37
TA	*L. decidua*	3	8.22-8.7	252.07-297.96	0.32-0.34
TA	*P. cembra*	1	6.45-6.45	409.35-409.35	0.27-0.27
KAS	*L. decidua*	4	10.34-10.79	434.64-475.42	0.37-0.44
KAS	*P. cembra*	3	9.98-10.91	439.76-473.75	0.4-0.44
KAN	*L. decidua*	2	9.73-10.01	417.33-431.83	0.36-0.37
KAN	*P. cembra*	2	10.79-11.02	438.33-472.32	0.44-0.45
EWLA (MJ)					
MAS	*L. decidua*	5	6.87-7.87	141.28-222.68	0.26-0.33
MAS	*P. cembra*	2	5.48-6.63	228.04-238.78	0.23-0.24
MAN	*L. decidua*	2	7.98-8.2	129.39-164.84	0.33-0.34
MAN	*P. cembra*	3	5.85-7.04	200.25-285.02	0.26-0.28
TA	*L. decidua*	3	4.86-6.46	130.23-176.87	0.26-0.3
TA	*P. cembra*	2	4.31-5.41	237.85-269.98	0.2-0.21
KAS	*L. decidua*	2	6.73-7.61	293.34-302.17	0.26-0.27
KAS	*P. cembra*	3	7.17-8.21	267.17-297.76	0.26-0.3
KAN	*L. decidua*	3	8.8-9.17	240.43-324.46	0.32-0.36
KAN	*P. cembra*	2	6.82-7.86	288.48-300.55	0.26-0.28

Climatic conditions were summarized over the May–July window for TRW (MJJ) and the May–June window for EWLA (MJ). The column *n* represents the number of positive pointer years detected for each proxy–site–species combination. Values represent the interquartile range (Q25–Q75) of climatic conditions observed during positive pointer years.

### 3.3 Long term growth trends and absolute growth

**Figure 6:**
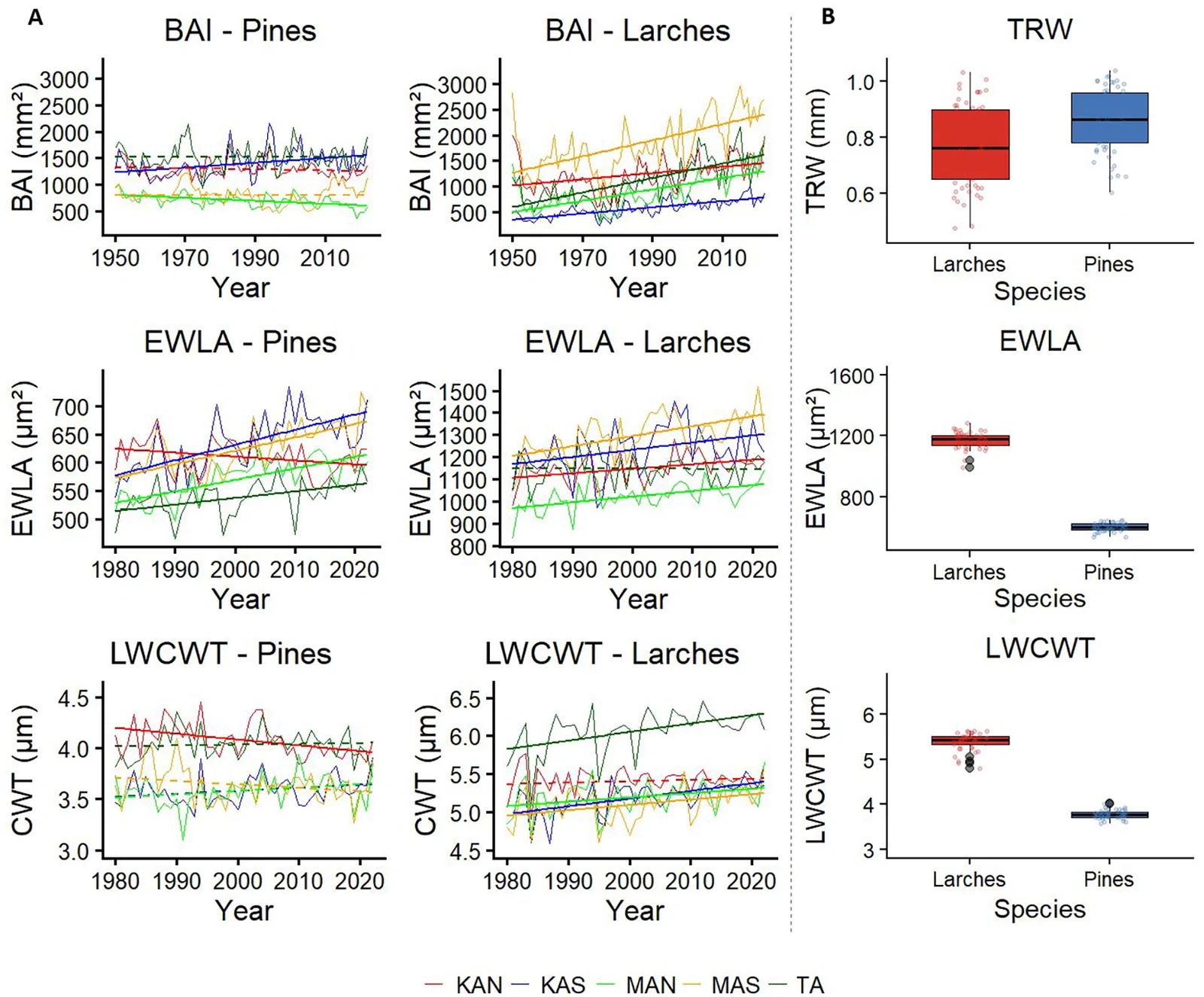
**(A)** Long-term growth trends for basal area increment (BAI; top, 1950-2022), earlywood lumen area (EWLA; middle, 1980-2022), and latewood cell wall thickness (LWCWT; bottom, 1980-2022) across sites and for both species. Analyses are based on raw (non-detrended) series to preserve long-term variability and highlight potential directional changes in growth. A subsample of trees with 150–300 rings was used to minimize potential age effects and homogenize datasets among sites (see Methods). Solid lines indicate statistically significant linear trends (p < 0.05), whereas dashed lines represent non-significant trends. Note that separate scales were used for y-axes to improve species-specific visualization of anatomical traits, because EWLA and LWCWT have markedly different absolute ranges. **(B)** Absolute values of tree-ring width (TRW; 1950-2022), EWLA (1980-2022), and LWCWT (1980-2022) for both species across all sites, for the same age-filtered subset. Boxplots represent the median (horizontal line), interquartile range (box), and 1.5× interquartile range whiskers; points indicate individual annual observations. Significant interspecific differences were detected for EWLA and LWCWT (ANOVA, p < 0.001), whereas TRW showed no significant difference.

Long-term growth trends differed between growth proxy, species, and site (**Figure 6A**). BAI trends of *P. cembra* were very variable across sites, with increasing, stable and decreasing trends, while BAI of *L. decidua* showed consistent and significant upward trends across all sites. EWLA increased significantly for both species at most sites. LWCWT showed stable trends at most sites for *P. cembra*, whereas for *L. decidua* it increased significantly at most sites (**Figure 6**). Tree-level GAMMs showed that long-term trajectories reflected a combination of ontogenetic, climatic and residual temporal components (**Supplementary Tables 6, 12**–**16**). For BAI, May–July temperature had a positive effect, while the positive effect of May–July VPD was larger in *L. decidua* than in *P. cembra* (+5.68% and +1.65% per +1 SD, respectively). May–July precipitation had a small negative effect, and additional negative winter and spring VPD effects were found only for *P. cembra*. For EWLA, tree height was the main positive fixed effect (+8.21% per +1 SD), while May–June climatic effects were weak, except for a small positive VPD effect in *L. decidua*. For LWCWT, July–August temperature and precipitation had positive effects, whereas VPD effects were not significant in either species. Residual temporal smooths indicated additional site-specific temporal dynamics, particularly for BAI.

Linear mixed-effects models revealed a significant species × growth proxy interaction, indicating that interspecific differences varied among TRW, EWLA and LWCWT (**Supplementary Table 18**). Estimated marginal means showed no significant difference in TRW between *P. cembra* and *L. decidua* (Holm-adjusted p = 0.197; **Figure 6B**, **Supplementary Table 19**). In contrast, both anatomical traits were significantly larger in *L. decidua* than in *P. cembra* (Holm-adjusted p < 0.001; **Figure 6B**, **Supplementary Table 19**). These patterns were unchanged in the sensitivity model including slope aspect (**Supplementary Table 20**).

## 4 Discussion

Our results revealed site- and species-specific differences in climate-growth responses of *L. decidua* and *P. cembra* in three subalpine treeline regions with differing moisture regimes. By integrating inter- and intra-annual analyses, we showed that *L. decidua* was more consistently associated with warmer conditions, as indicated by mostly positive temperature-growth responses and wider TRW during warmer years. In contrast, *P. cembra* appeared more constrained by moisture availability, especially at the drier sites, as reflected by stronger precipitation and VPD responses and wetter conditions associated with positive TRW and EWLA. Long-term growth trajectories showed a more complex picture: although trends differed between species and sites, the GAMM analyses indicated that effects were only partly attributed to climate, while ontogenetic, tree-level, and residual temporal components played a considerable role as well.

### 4.1 Species-specific climate-growth responses

As expected, both species showed significantly positive temperature-growth correlations for TRW and LWCWT during the growing season at most sites (Leonelli et al., 2009; Frank and Esper, 2005; Carrer et al., 2007; Büntgen et al., 2007). Low temperatures constrain the duration and rate of xylogenesis in cold treeline environments, whereas warmer spring-summer conditions advance cambial onset, enhance tracheid production, and accelerate differentiation, thus widening rings (Körner, 2012; Rossi et al., 2007, 2008) and thickening walls (Cuny and Rathgeber, 2016; Carrer et al., 2018). Surprisingly, the expected positive relationship between temperature and tree growth was weak at the drier Mazia sites (MAS, MAN, partly TA) for *P. cembra*, possibly due to the gap between the current realized upper tree limit (~2300 m a.s.l.) and the climatic treeline isotherm caused by historical grazing (Obojes et al., 2022). In contrast, temperature effects were stronger at the more humid Karwendel sites and on north-facing slopes, where cooler, moister microclimates can amplify temperature limitation of growth (Carrer et al., 2007, but see Kuželová et al., 2025). Overall, *L. decidua* showed a stronger temperature-growth response than *P. cembra* particularly in LWCWT, indicating a stronger common site signal, which is also reflected in its higher values of chronology statistics (rbar and EPS, **Supplementary Table 3**).

At the drier inner-alpine sites (MAN, MAS, TA), *P. cembra* TRW was sensitive to spring precipitation (positive) and VPD (negative), consistent with our H1 and other Alpine *P. cembra* networks (Saulnier et al., 2011; Leonelli et al., 2011). Higher spring precipitation may increase snowpack and soil-water recharge before cambial reactivation, promoting wider rings (Fonti et al., 2010; Gruber et al., 2009). At TA, TRW of both species showed a strong positive summer temperature response that was largely absent in EWLA and LWCWT for *P. cembra*, suggesting that TRW mainly reflected increased tracheid number in response to temperature (Cuny and Rathgeber, 2016; Cuny et al., 2018; Cuny et al., 2014; Fonti et al., 2010; Unterholzner et al., 2024). This contrasts with Lopez-Saez et al. (2023), who found a positive LWCWT-temperature relationship for *P. cembra* at a geographically close site. The discrepancy may reflect temporal differences (1920–2017 vs. 1980-2022), with warmer recent decades possibly increasing evaporative demand and offsetting latewood thickening, or differences in sample replication.

EWLA responses to climate were weaker and more variable, consistent with the multifactorial control of cell enlargement (Baison et al., 2020; Zheng et al., 2021). As hypothesized, *L. decidua* showed positive EWLA-temperature associations in spring-summer at most sites, suggesting that warmer growing seasons enhance tracheid enlargement through accelerated cambial activity when water potential remains adequate (Fonti et al., 2010; Cuny et al., 2018; Carrer et al., 2017). At MAS, MAN and TA, *L. decidua* EWLA additionally correlated positively with current summer precipitation, underscoring the importance of water availability for cell enlargement of this species (Gruber et al., 2009). In Karwendel (KAN, KAS), the negative correlation between *P. cembra* EWLA and winter temperature may indicate that warmer winters reduce chilling accumulation and delay or desynchronize dormancy release, thereby compressing the early-season enlargement phase. Because final tracheid size largely depends on the duration of cell enlargement, a shortened enlargement period can translate into a smaller lumen area (Asse et al., 2018; Cuny et al., 2014).

Generally, these findings align with *P. cembra*’s isohydric behavior, where early stomatal closure under atmospheric drought limits tracheid enlargement and lumen size (Anfodillo et al., 1998; Wieser, 2012; Știrbu et al., 2022; Unterholzner et al., 2024). Conversely, *L. decidua*’s anisohydric strategy allows for continued enlargement under warmer conditions, producing larger lumina and a temperature-driven EWLA signal (Carrer et al., 2017; Cuny et al., 2018). Nevertheless, caution is needed when interpreting climate–anatomy relationships in our study, as their weaker common signal may increase uncertainty around site-specific responses (**Supplementary Table 3**; Jourdain et al., 2025).

### 4.2 Site-, species- and proxy-specific positive growth departures in years with thermal and hydroclimatic anomalies

The pointer-year analysis provided an event-based view of the site-, species- and proxy-specific climate responses observed in the correlation analyses. For TRW, positive pointer years were synchronized between species in 1982, 1983 and 1994, suggesting that these years were broadly favorable for radial growth across regions. However, EWLA showed a different temporal pattern, and we only found five shared TRW and EWLA events overall. This limited overlap between proxies suggests different drivers of their growth and is plausible because TRW integrates wood formation over the entire growing season, whereas EWLA more directly reflects early-season tracheid enlargement (Fonti et al., 2010; Cuny et al., 2014; Eilmann et al., 2011). Frequency and magnitude of pointer years were generally similar between proxies, suggesting that EWLA could serve as an equally good proxy of extreme climatic conditions as TRW.

The climatic conditions associated with these positive pointer years very clearly show the contrasting ecological strategies of the two species. In *P. cembra*, positive TRW pointer years at the drier MA and TA sites were mainly associated with a wetter-than-average growing season, and positive wider EWLA occurred in cooler and lower-VPD growing seasons, with additional links to precipitation at the drier sites. This pattern suggests that above-average total radial growth and earlywood enlargement in *P. cembra* are partly linked to reduced atmospheric drought and greater water availability, particularly at the drier sites, consistent with the species’ conservative water-use strategy and sensitivity to moisture limitation (Anfodillo et al., 1998; Wieser, 2012; Obojes et al., 2022). In contrast, *L. decidua* consistently formed wider rings during years with a warmer and drier growing season, consistent with its stronger temperature-growth response and a greater capacity to maintain growth under warmer but drier conditions (Saderi et al., 2019; Saulnier et al., 2019; Cuny et al., 2018). In line with the correlation analysis, its EWLA responses to temperature and climate in general were weaker, suggesting other drivers of earlywood lumen enlargement.

Climatic envelopes show that exceptionally wide rings occurred under lower temperature, precipitation, and VPD ranges at the drier and colder MAS–MAN–TA sites than at the wetter KA sites, suggesting that favorable radial growth was defined by local climatic baselines rather than uniform thresholds.

Similarly and consistent with our other observations, temperature and VPD ranges associated with positive pointer years were generally lower for *P. cembra*, especially for EWLA, suggesting that large earlywood and TRW growth occurred under comparatively low evaporative demand rather than warm conditions alone. In contrast, the lower precipitation ranges associated with positive EWLA pointer years in *L. decidua* suggest a weaker dependence on wet conditions, consistent with its lower sensitivity to moisture limitation (Cuny et al., 2018; Rozenberg et al., 2020; Tumajer et al., 2025).

Thus, our hypothesis was partially supported: positive pointer years confirmed species-specific climatic controls, with *P. cembra* more closely linked to wetter or lower-VPD conditions and *L. decidua* TRW more closely linked to warmer conditions.

### 4.3 Growth trends

Growth trajectories of BAI diverged between species: In *L. decidua*, the positive BAI trends are consistent with positive effects of May–July temperature and VPD in the GAMM. This suggests that *L. decidua* may have benefited from warmer and drier growing seasons, as expected in generally cold-limited environments. In contrast*, P. cembra* showed positive, stable and negative BAI trends despite a comparable age structure. Although May–July temperature and VPD had positive effects in the model, these were partly counterbalanced by negative winter and spring VPD effects, suggesting that higher atmospheric dryness outside the main growing-season window may have contributed to weaker or declining BAI trajectories in this species. However, the GAMM results suggest that these patterns cannot be attributed to climate alone, because BAI trajectories were associated with a combination of climatic, ontogenetic, tree-specific and residual temporal components (**Supplementary Tables 12**-**15**).

Trends in LWCWT further support contrasting growth strategies between the two species (**Figure 6**). In *L. decidua*, LWCWT increased at almost all sites, consistent with the positive effect of July–August temperature and the moderate fitted climatic amplitude of the LWCWT model. This is in line with warmer late-summer conditions enhancing wall-thickening processes and carbon investment into structural tissues (Fonti et al., 2010; Cuny and Rathgeber, 2016). In contrast, *P. cembra* showed mostly stable LWCWT trends. Our results suggest that late-season warming did not translate into a consistent long-term increase in LWCWT across *P. cembra* sites, despite the common positive effect of July–August temperature in the model (**Supplementary Tables 10**–**16**). This may indicate that the temperature signal was partly modulated by site-specific temporal dynamics and individual-tree variability, although previous studies have shown that cell-wall thickness in *P. cembra* can be strongly temperature-sensitive (Carrer et al., 2018; López-Sáez et al., 2023; Știrbu et al., 2022). Overall, our findings support the view that radial growth and wood anatomy of *P. cembra* is less able to benefit from warm conditions when this is not accompanied by sufficient moisture availability, possibly due to its higher drought sensitivity and more conservative physiology, whereas *L. decidua* displays more positively responsive growth under favorable thermal conditions (Obojes et al., 2022; Știrbu et al., 2022).

Contrary to our expectations, EWLA increased in both species at most sites, indicating that H3 is only partially supported. The EWLA model showed that May–June climatic effects were weak, whereas tree height was positively associated with EWLA and individual-tree variability was substantial. Thus, increasing EWLA likely reflects primarily structural and allometric changes associated with tree height and increasing conduit dimensions over time (Carrer et al., 2015; Anfodillo et al., 2016), even though only mature trees (> 150 rings) were analyzed here.

### 4.4 Interspecific differences in absolute radial growth and wood anatomical traits

Linear mixed-effects models showed no significant interspecific difference in TRW but significantly higher EWLA and LWCWT in *L. decidua* than in *P. cembra* (**Figure 6B**; **Supplementary Tables 18**, **19**), in agreement with findings of other studies (Rossi et al., 2009; Cartenì et al., 2018). Significant differences in the dimensions of wood anatomical traits, despite similar radial increment, suggest fundamentally different ring construction and ecophysiological regulation strategies between *P. cembra* and *L. decidua*.

A greater EWLA generally reflects higher hydraulic efficiency (Sperry et al., 2008), while increased LWCWT enhances mechanical reinforcement of the xylem and resistance to conduit implosion under negative water potentials (Hacke et al., 2001; Pittermann et al., 2006). Taken together, the anatomical configuration observed in *L. decidua* suggests prioritization of conduit efficiency and structural reinforcement (Sperry et al., 2008; Hacke et al., 2001). This is in line with our other observations and with this fast-growing pioneer often being characterized by a less conservative stomatal behavior that may sustain stronger cell enlargement and wall thickening during favorable thermal periods (Anfodillo et al., 1998; Wieser, 2012; Cuny and Rathgeber, 2016). In contrast, *P. cembra* is generally described as more conservative in its water-use behavior, potentially limiting turgor-driven cell enlargement during periods of elevated vapor pressure deficit (Anfodillo et al., 1998). Under such conditions, cambial activity may continue while cell expansion remains constrained, leading to similarly wide rings but composed of a larger number of smaller tracheids with thinner cell walls (Cuny et al., 2014).

Importantly, these findings provide a mechanistic context for the long-term growth patterns of both species observed in this study and in previous research (e.g., Obojes et al., 2022; Cartenì et al., 2018; Rossi et al., 2009). By combining the analyses of radial growth and wood anatomical tree-ring proxies, our findings support the interpretation of interspecific differences in ring construction, critical for understanding tree growth responses to climate change (Fonti et al., 2010; Cuny et al., 2014; Cuny et al., 2018).

### 4.5 Scope of inference, limitations, and future research directions

Our findings should be interpreted within the scope of the sampling design and analytical framework. First, the quantitative wood anatomical dataset included a limited number of trees per site and species, reflecting the labor-intensive nature of this approach. Consequently, inference for individual site × species chronologies is constrained, especially where the common signal is weaker, supporting our focus on consistent patterns across sites, species, and traits.

Second, this study provides robust observational evidence from long-term tree-ring and anatomical records. Tree-level GAMMs helped separate structural/ontogenetic, climatic, individual-tree, and residual temporal components, strengthening mechanistic interpretation, although causal relationships cannot be established directly. Accordingly, the inferred divergent growth trajectories of *L. decidua* and *P. cembra* should be regarded as evidence-based hypotheses rather than direct predictions of future competitive dynamics or stand composition.

Third, inference is restricted to our five study sites spanning contrasting hydroclimatic conditions. Although these capture important environmental gradients, factors such as soil properties, competition, disturbance history, snow regime, recruitment, and demography were not explicitly considered and may influence future species performance.

Future research should expand anatomical sampling across sites and time, increase environmental coverage, and integrate long-term monitoring with experimental approaches under controlled conditions. Combining quantitative wood anatomy with xylogenesis, dendrometer, stable isotope, sap-flow, and *in-situ* weather measurements would further strengthen understanding of climate-growth mechanisms and improve projections of species responses to future warming and atmospheric drought.

## 5 Summary and conclusions

Our holistic assessment of radial growth and wood anatomical traits of *L. decidua* and *P. cembra* growing in treeline ecotones of the European Alps revealed species-, site-, and trait-specific differences in their response to climate: In addition to temperature, precipitation and atmospheric drought (VPD) emerged as significant drivers of growth variability, with *P. cembra* showing a stronger dependence on moisture particularly at drier sites, and *L. decidua* responding more consistently to warmth. The assessment of long-term growth trajectories and absolute growth further underscored the contrasting strategies of the two species: along with hydraulically efficient wood anatomical structures, *L. decidua* showed increases in growth, whereas the more conservative *P. cembra* exhibited variable growth trends, partly dependent on site climatic conditions. These findings support the hypothesis that continued warming and increasing atmospheric drought may contribute to divergent growth trajectories of *L. decidua* and *P. cembra* at Alpine treeline ecotones, particularly where moisture availability becomes increasingly limiting. Future research combining larger anatomical datasets, longer monitoring periods, broader site networks, and integrated empirical–experimental approaches will be needed to test this hypothesis, strengthen causal inference, and account for environmental conditions and stand dynamics.

## Data Availability

The raw data supporting the conclusions of this article will be made available by the authors, without undue reservation.
