# Supplementary material for: Divergent growth and wood anatomical responses of European larch and Swiss stone pine to climate variability at alpine treeline ecotones
Source: Front Plant Sci. 2026 Aug 6;17:1813904. doi: 10.3389/fpls.2026.1813904 (PMC13490113; doi:10.3389/fpls.2026.1813904)
Supplement: Supplementary file 1 [file DataSheet1.pdf]

SUPPLEMENTARY MATERIAL

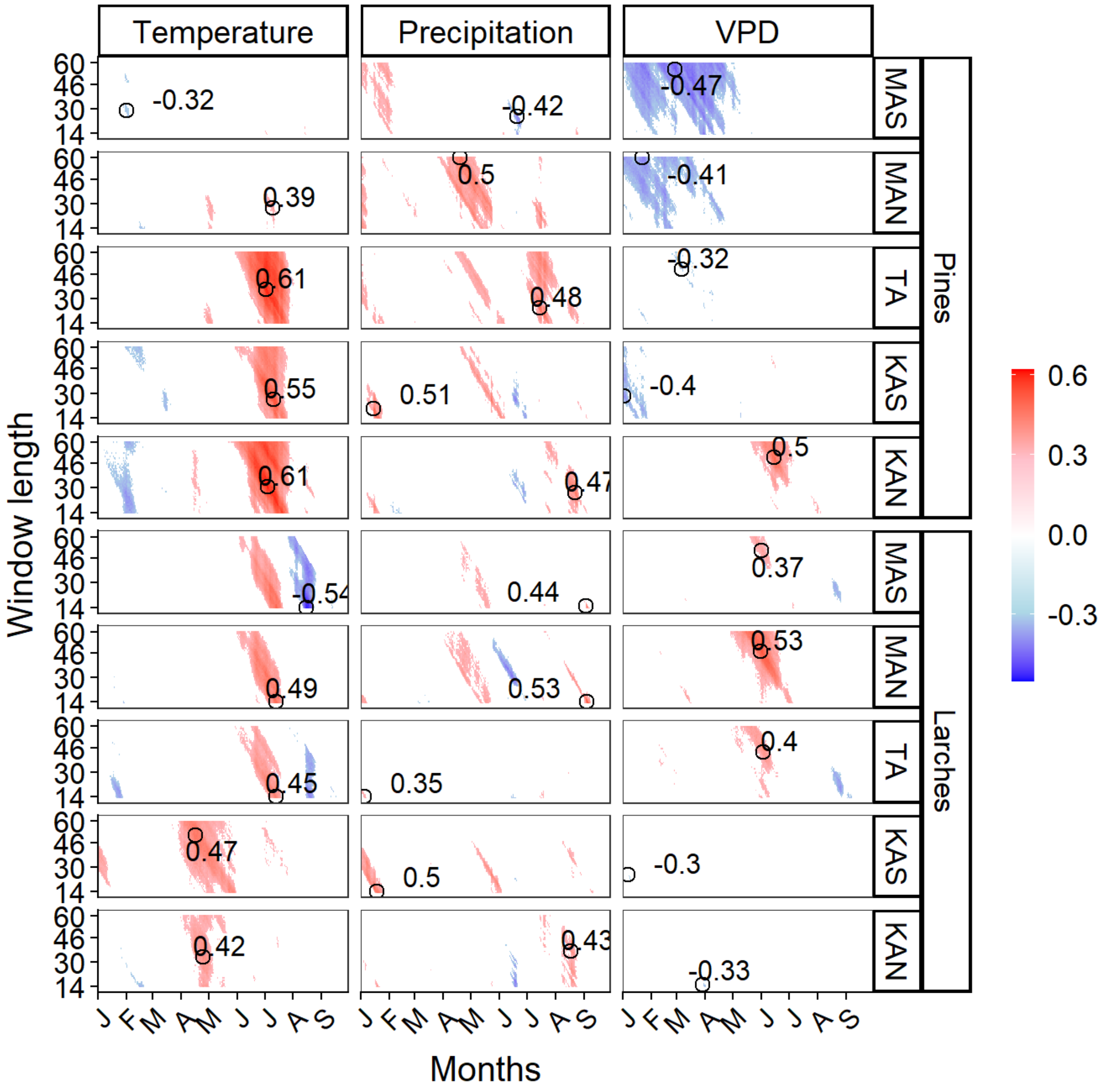

Figure S1. Partial bootstrap correlations between non detrended daily climate data (mean, minimum, maximum temperature) and tree-ring width. Letters on x axis indicate the months of the current year from January to September. The correlation window ranges from 14 to 60 consecutive days (y axis). Only significant correlations are shown (p<0.05). The strongest significant correlation of each subplot is indicated by a circle and the respective value.

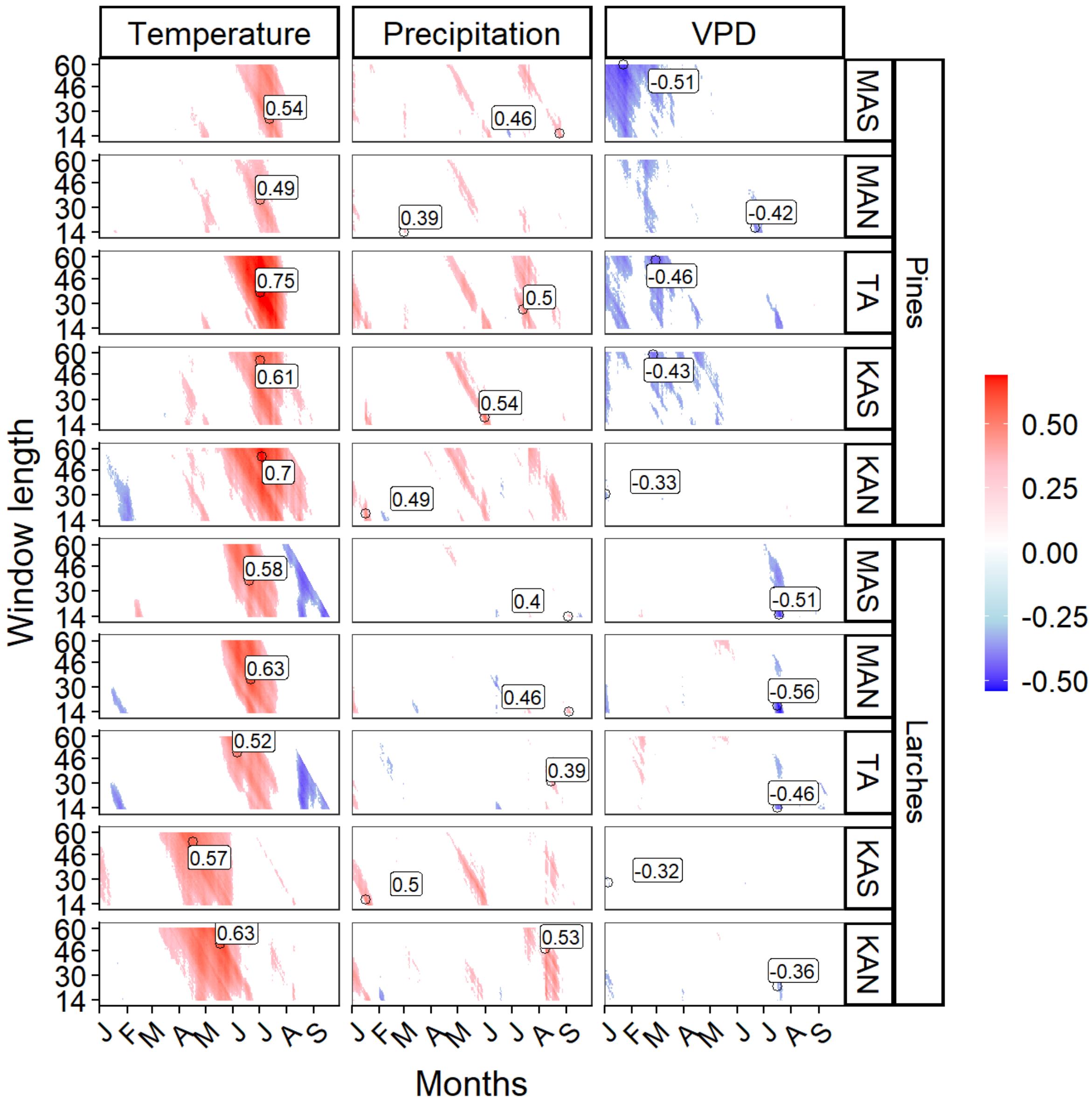

Figure S2. Partial bootstrap correlations between detrended daily climate data (mean, minimum, maximum temperature) and detrended tree-ring width (10 years spline). Letters on x axis indicate the months of the current year from January to September. The correlation window ranges from 14 to 60 consecutive days (y axis). Only significant correlations are shown (p<0.05). The strongest significant correlation of each subplot is indicated by a circle and the respective value.

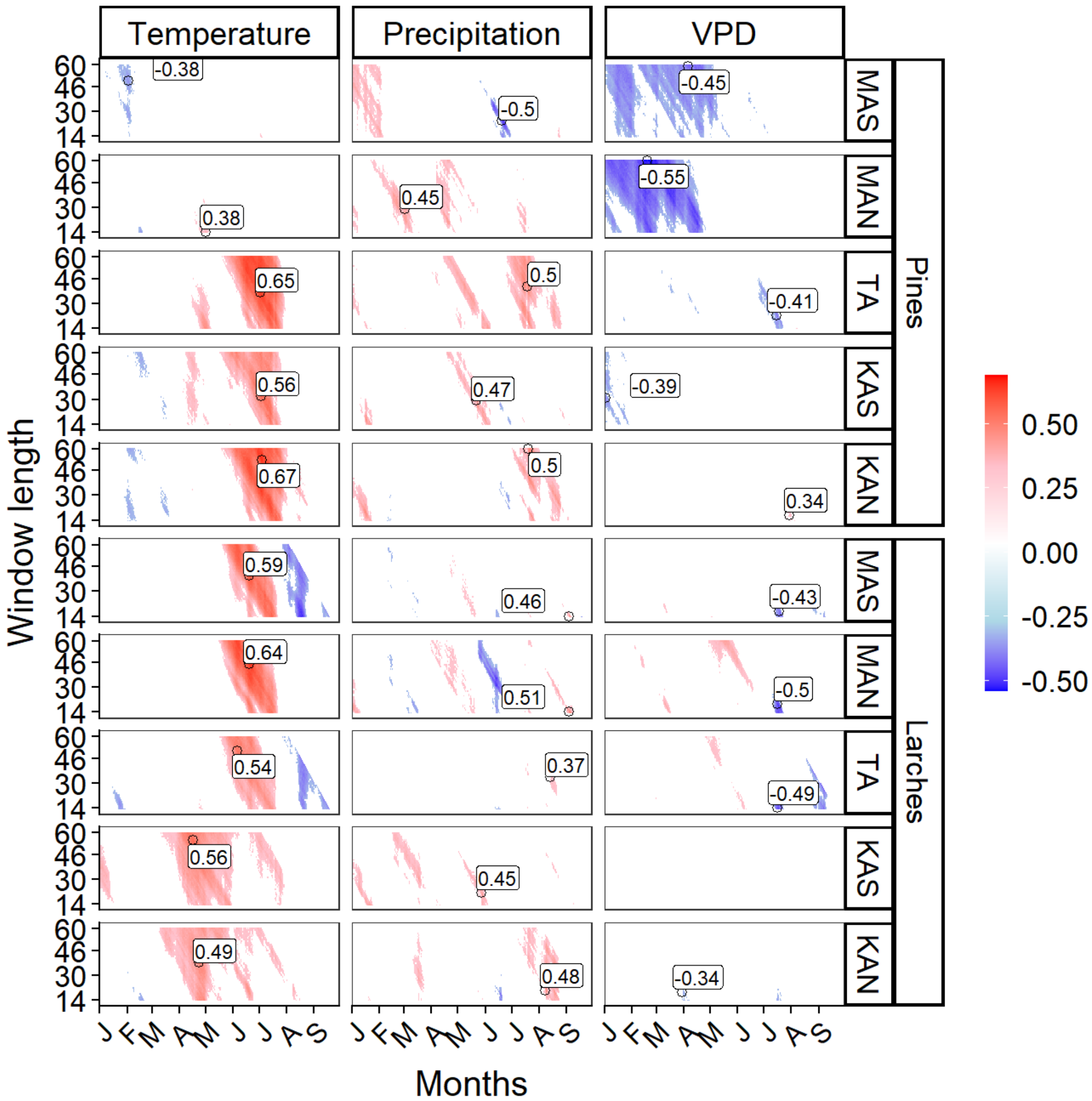

Figure S3. Partial correlations between detrended daily climate data (mean, minimum, maximum temperature) and detrended tree-ring width (70 years spline). Letters on x axis indicate the months of the current year from January to September. The correlation window ranges from 14 to 60 consecutive days (on y axis). Only significant correlations are shown ($p<0.05$). The strongest significant correlation of each subplot is indicated by a circle and the respective value.

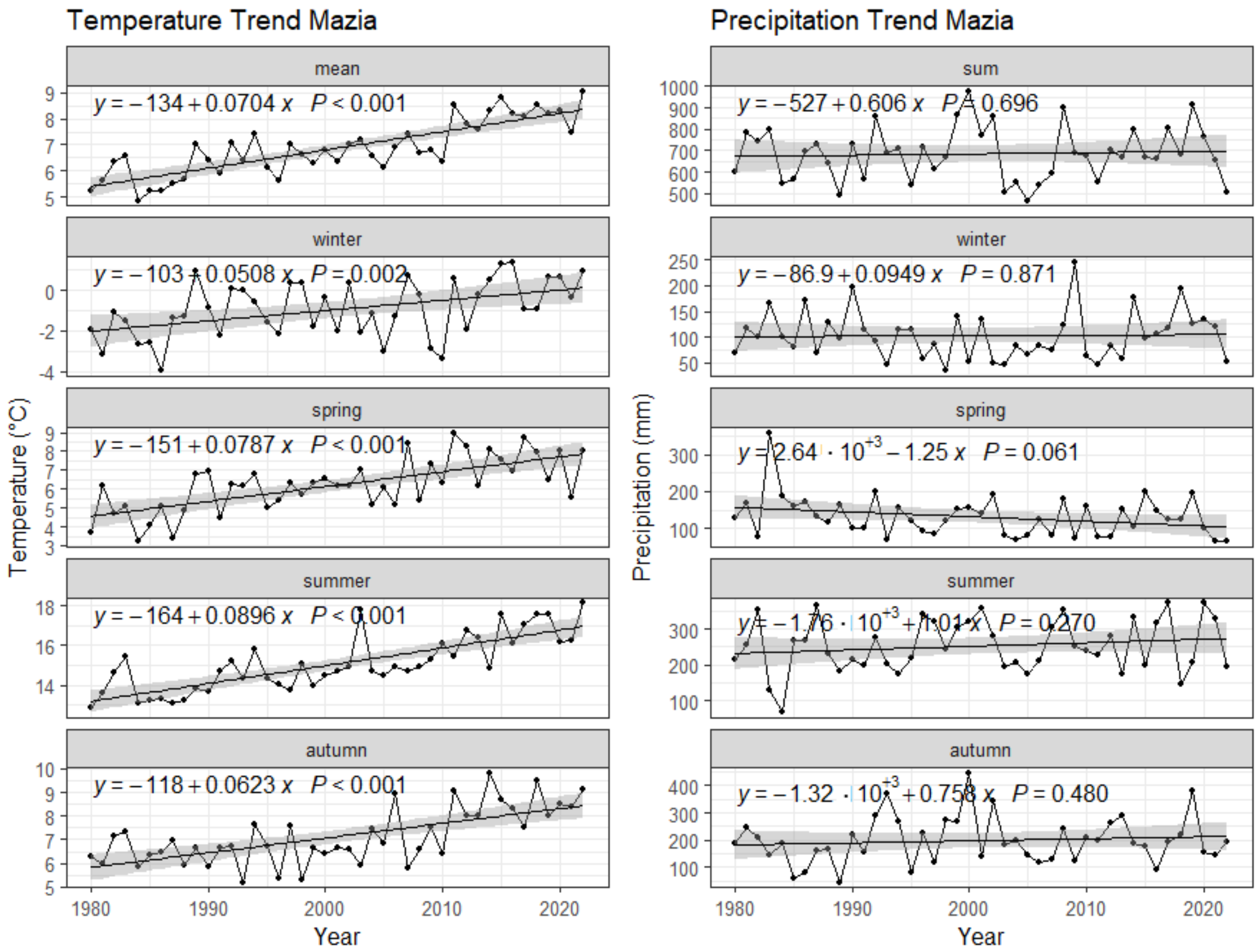

Figure S4. Temperature and precipitation trends for 1980-2022 from the Monte Maria meteorological station (~ 1300 m), close to the Mazia site. Seasons were defined as follows: winter (December-February), spring (March-May), summer (June-August), and autumn (September-November).

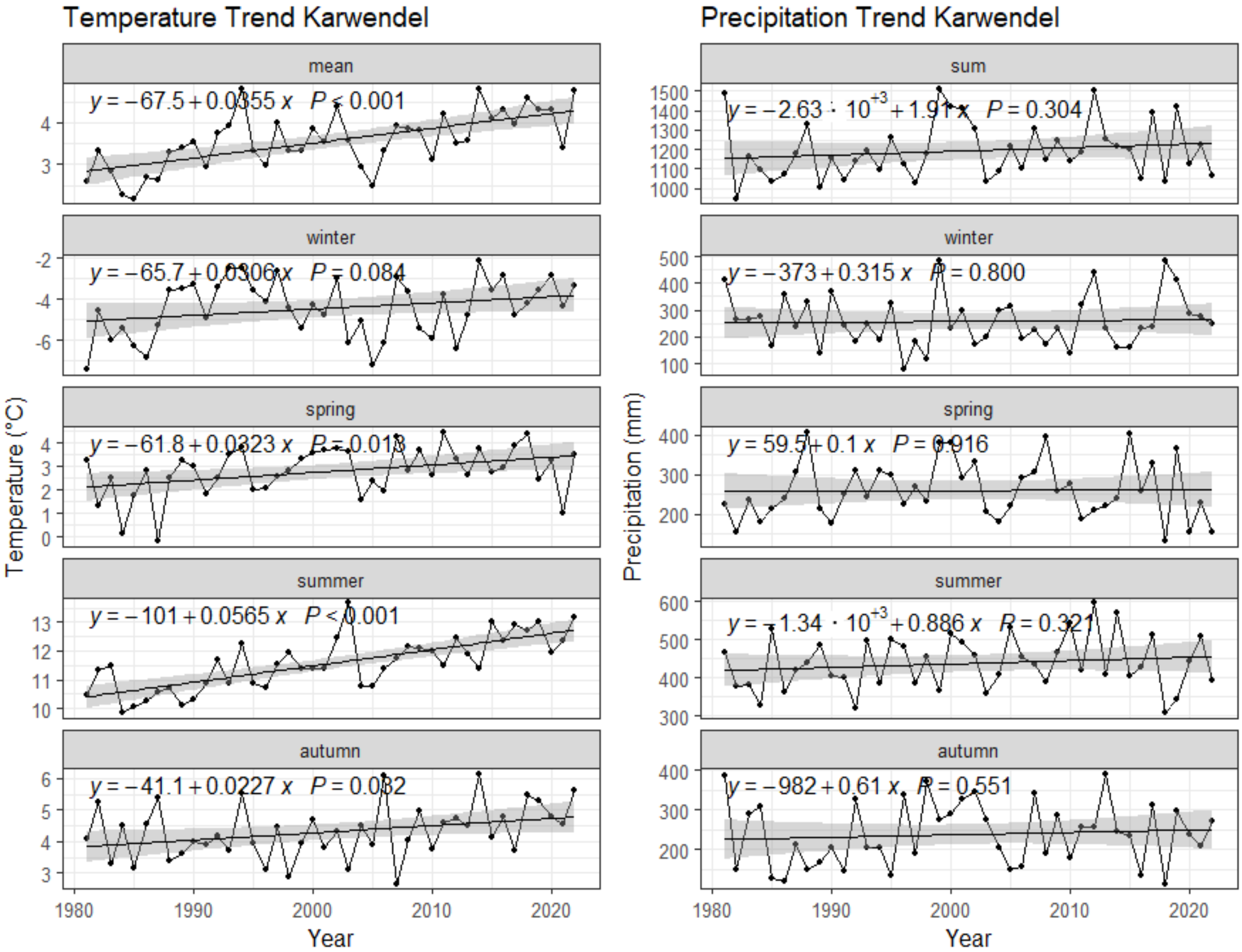

Figure S5. Temperature and precipitation trends for 1980-2022 from the Seefeld meteorological station (~ 1200 m), close to the Karwendel site. Seasons were defined as follows: winter (December-February), spring (March-May), summer (June-August), and autumn (September-November).

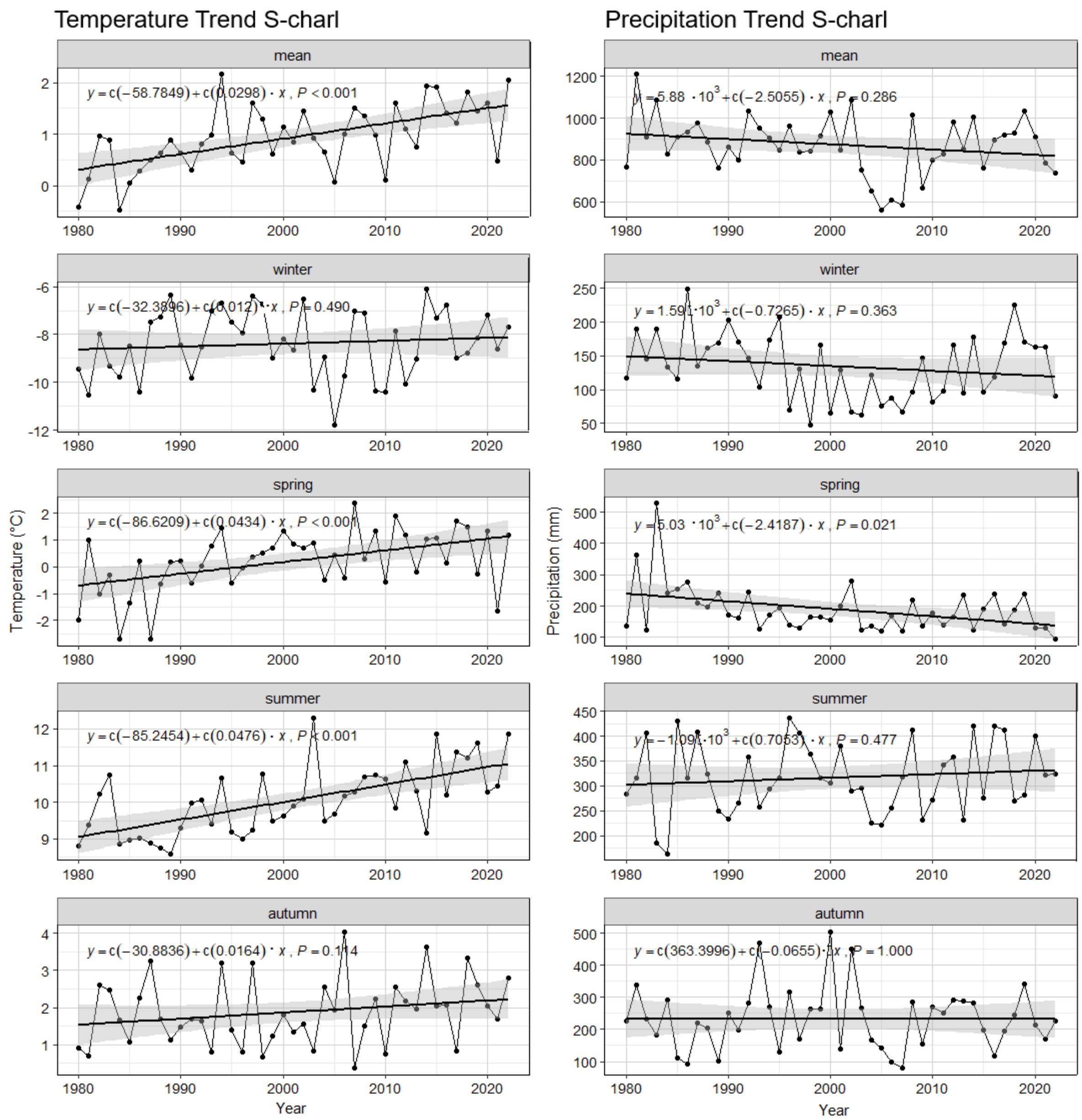

Figure S6. Temperature and precipitation trends for 1980-2022 from Buffalora meteorological station (~ 2000 m), close to the S-charl site. Seasons were defined as follows: winter (December-February), spring (March-May), summer (June-August), and autumn (September-November).

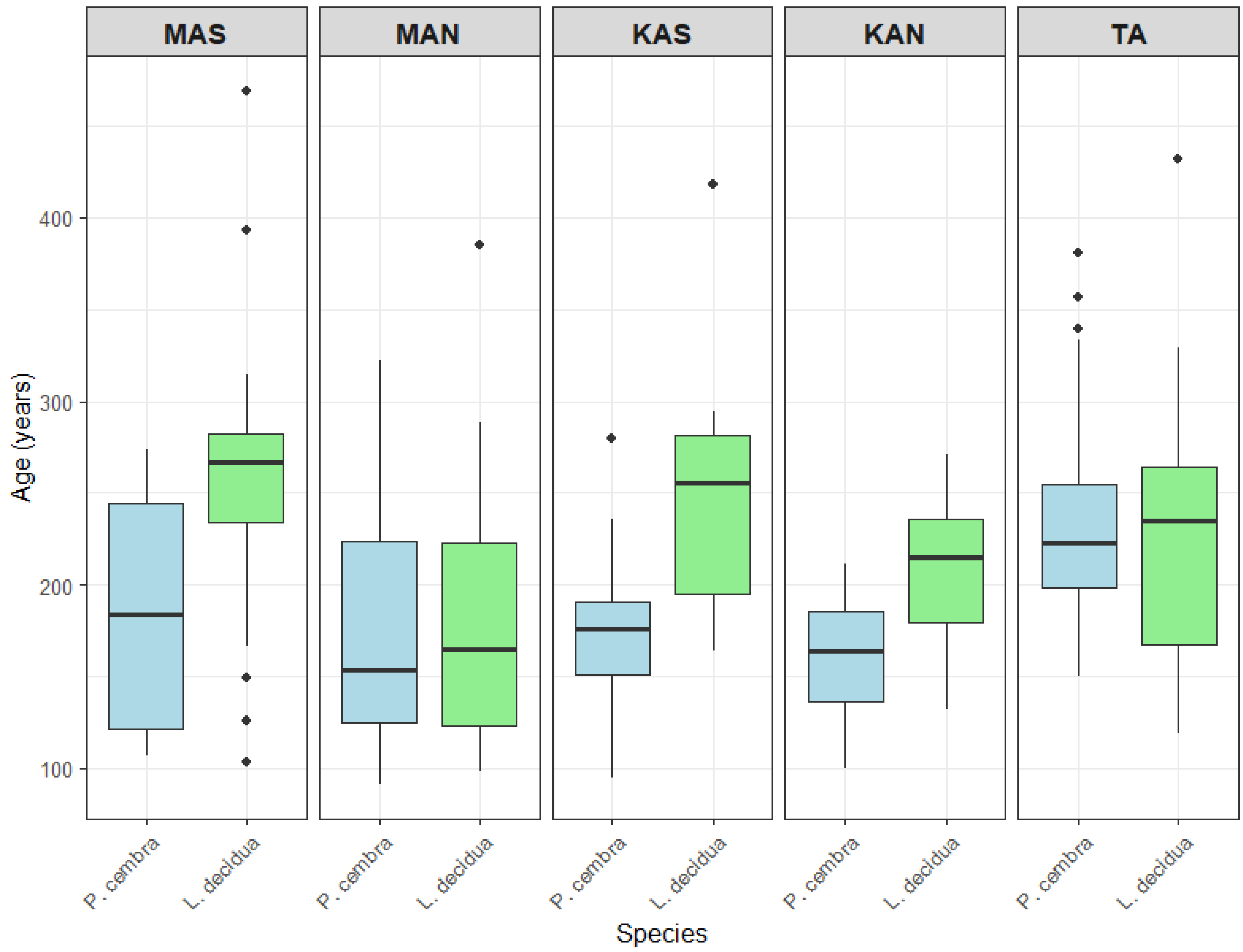

Figure S7. Boxplots of age distribution for *P. cembra* and *L. decidua*.

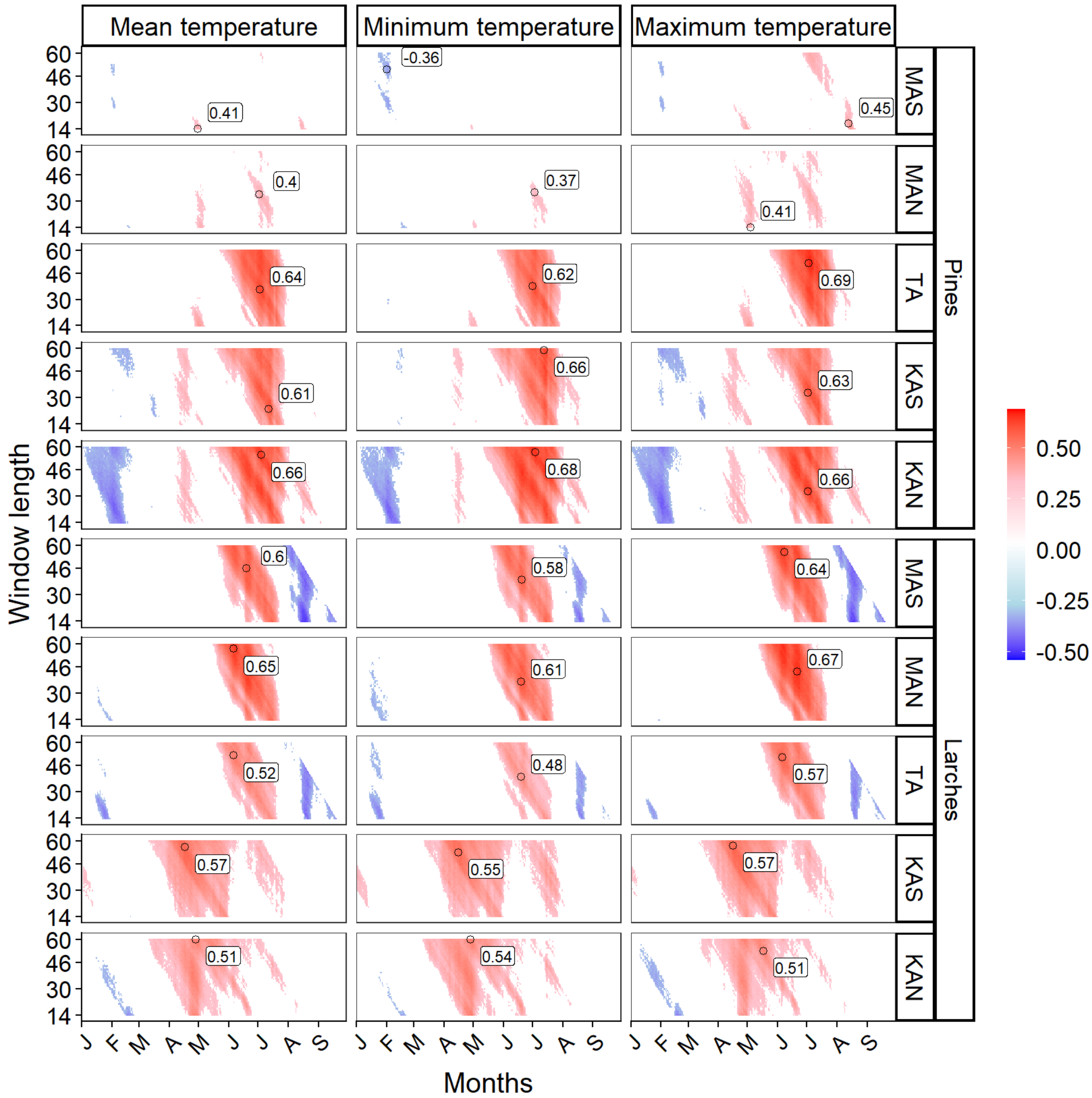

Figure S8. Partial bootstrap correlations between detrended daily climate data (mean, minimum, maximum temperature) and tree-ring width. Letters on x axis indicate the months of the current year. The correlation window ranges from 14 to 60 consecutive days (on y axis). Only significant correlations are shown (p<0.05). The strongest significant correlation of each subplot is indicated by a circle and the respective value.

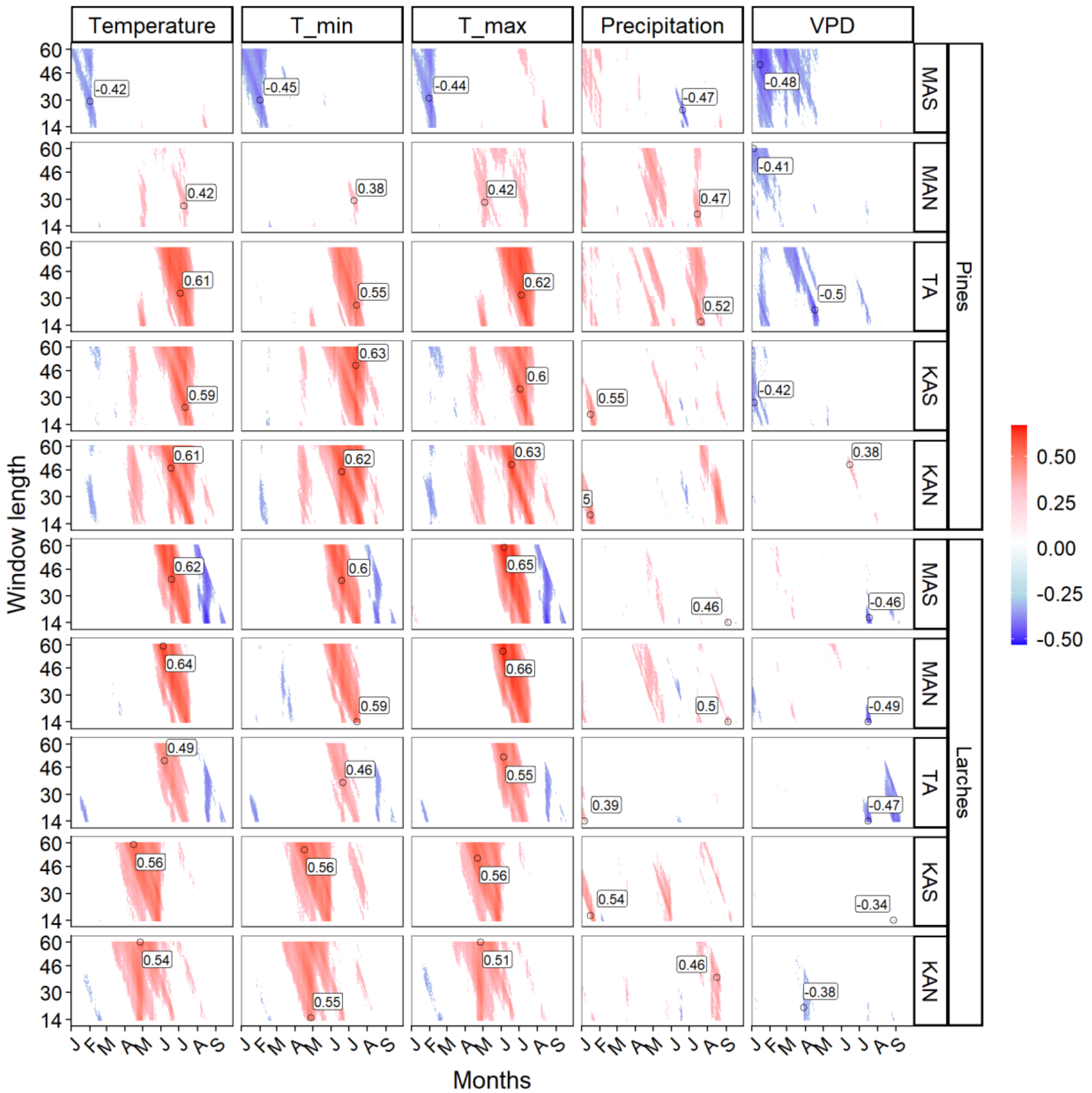

Figure S9. Partial bootstrap correlations between detrended daily climate data (mean, maximum, minimum temperature, total precipitation, and VPD) and Basal area increment. Letters on x axis indicate the months of the current year. The correlation window ranges from 14 to 60 consecutive days (on Y axis). Only significant correlations are shown (p<0.05). The strongest significant correlation of each subplot is indicated by a circle and the respective value.

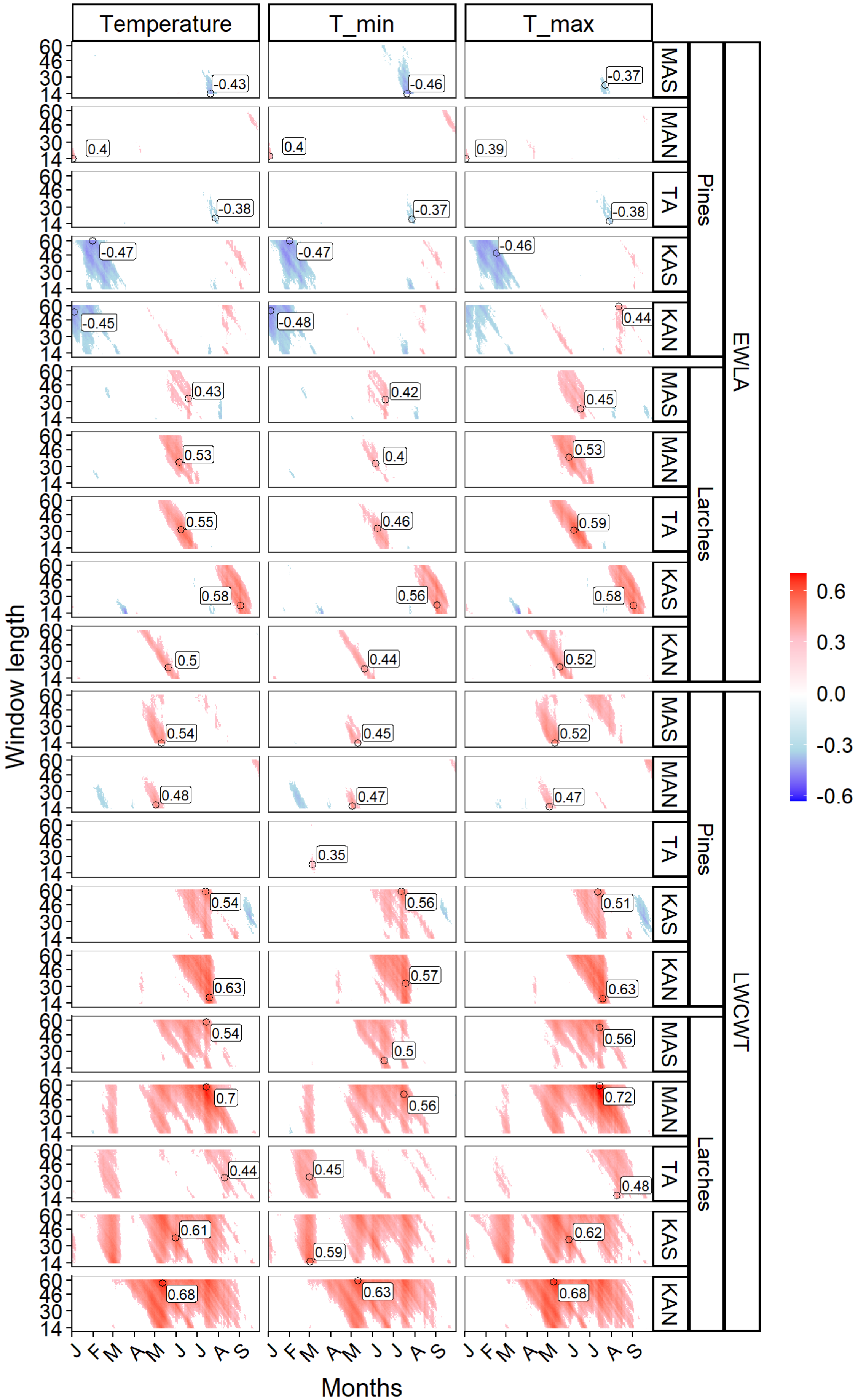

Temperature
T_min
T_max
MAS
MAN
TA
KAS
KAN
Pines
Larches
EWLA
LWCWT
Window length
60
46
30
14
J F M A M J J A S
Months
0.6
0.3
0.0
-0.3
-0.6

Figure S10. Partial correlations between detrended daily climate data (mean, minimum, maximum temperature) and earlywood lumen area and latewood cell wall thickness. Letters (on x axis) indicate the months of the current year. The correlation window ranges from 14 to 60 consecutive days (on y axis). Only significant correlations are shown (p<0.05). The strongest significant correlation of each subplot is indicated by a circle and the respective value.

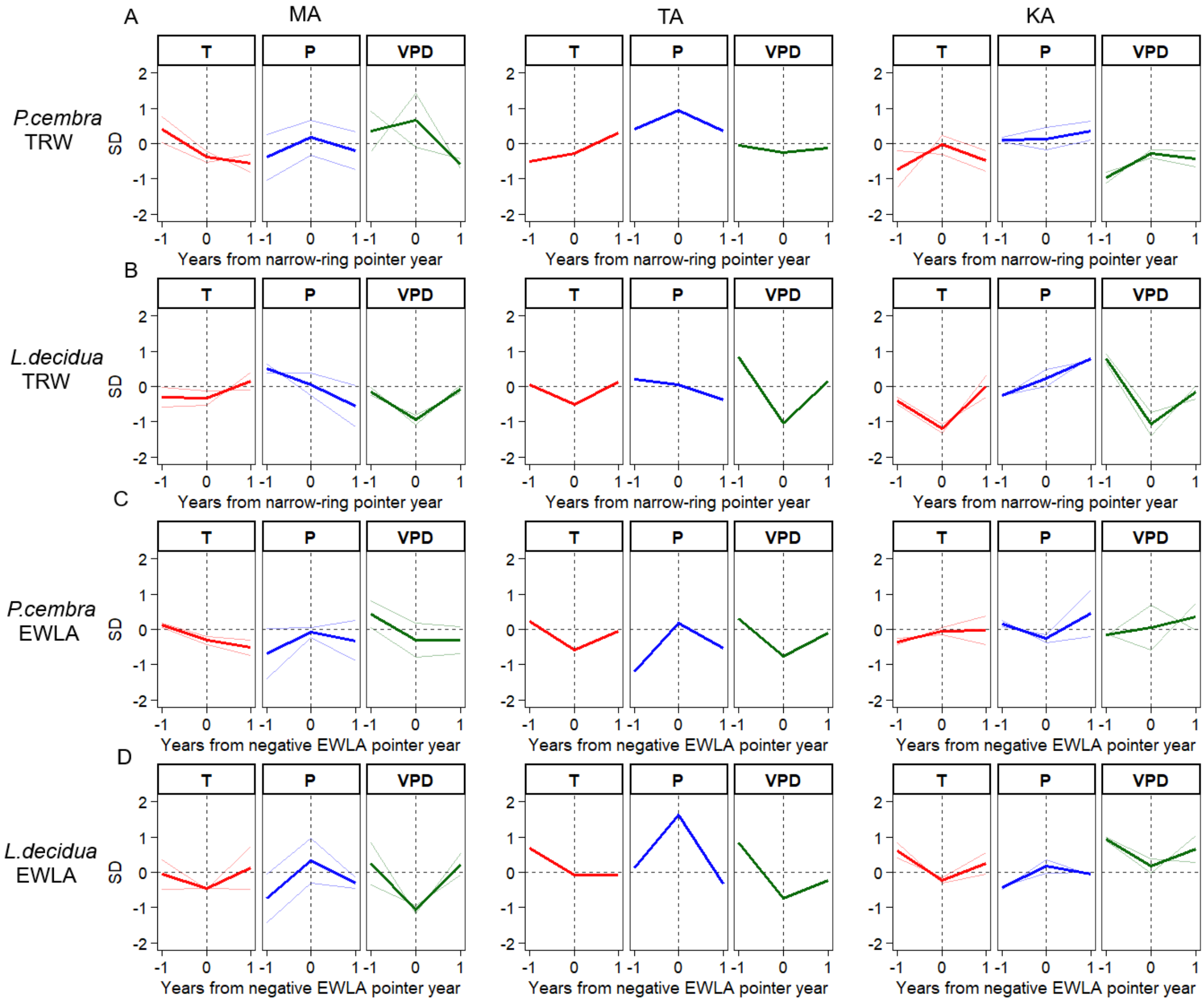

Figure S11. Climate anomalies associated with exceptionally narrow tree rings and narrow earlywood lumen area (EWLA) during negative pointer years. Negative pointer years were identified from standardized tree-ring width (TRW; panels A, B) and EWLA chronologies (panels C, D) for *P. cembra* (A, C) and *L. decidua* (B, D), using a threshold of -1.5 SD below the long-term mean. Standardized anomalies of mean temperature (T), precipitation sums (P), and vapour pressure deficit (VPD) are shown from one year before (-1) to one year after (+1) the pointer year (0). Climate variables were calculated over fixed seasonal windows: May–July for TRW and May–June for EWLA. Columns represent the three study regions: Mazia (MA), S-charl (TA), and Karwendel (KA). Thin lines indicate site-level climate anomalies, while thick lines represent the regional mean response. Horizontal dashed lines indicate zero climate anomaly, and vertical dashed lines mark the negative growth pointer year.

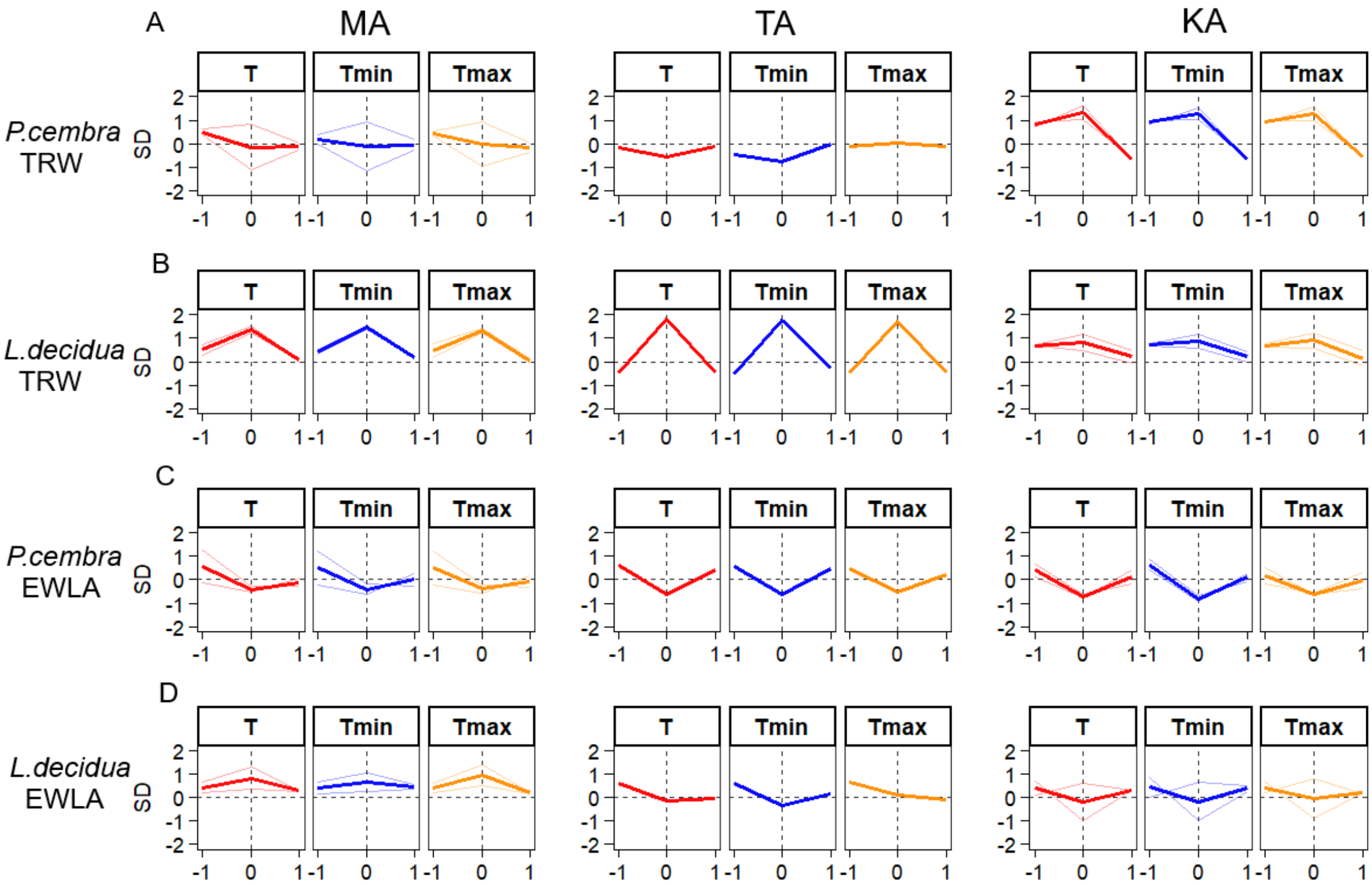

Figure S12. Temperature-dependent (mean, min., max.) pointer years in TRW (A,B) and EWLA (C,D) for *P. cembra* (A,C) and *L. decidua* (B,D) across the three study regions: Mazia (left), Karwendel (middle), and S-charl (right). Years with exceptionally wide or narrow rings were identified as values exceeding ±1.5 standard deviations from the mean, highlighting periods of anomalously favourable or unfavourable growth in relation to extreme climatic conditions.

Climate anomalies associated with exceptionally wide or narrow tree rings and earlywood lumen area (EWLA) during pointer years. Pointer years were identified from standardized tree-ring width (TRW; panels A, B) and EWLA chronologies (panels C, D) for *P. cembra* (A, C) and *L. decidua* (B, D), using a threshold of +/-1.5 SD below the long-term mean. Standardized anomalies of mean, maximum and minimum temperature are shown from one year before (-1) to one year after (+1) the pointer year (0). Climate variables were calculated over fixed seasonal windows: May–July for TRW and May–June for EWLA. Columns represent the three study regions: Mazia (MA), S-charl (TA), and Karwendel (KA). Thin lines indicate site-level climate anomalies, while thick lines represent the regional mean response. Horizontal dashed lines indicate zero climate anomaly, and vertical dashed lines mark the pointer year.

Table S1. Basic site characteristics and quality statistics for tree-ring width (TRW) site chronologies for the study period 1950-2022. RBAR represents the mean inter-series correlation, while EPS (Expressed Population Signal) indicates the robustness of each chronology in representing the population signal.

SITE	COORDINATES	ELEVATION	SPECIES	N. TREES	RBAR	EPS	MEAN AGE	MEAN TRW
MAS	46°41′N, 10°39′E	2300	PINES	19	0.423	0.933	184	0.87
MAN	46°44′N, 10°40′E	2250	PINES	20	0.41	0.933	175	0.90
KAS	47°21′N, 11°28′E	1900	PINES	20	0.378	0.927	174	1.03
KAN	47°21′N, 11°22′E	1700	PINES	18	0.334	0.905	160	1.06
TA	46°42′N, 10°20′E	2250	PINES	20	0.415	0.934	240	0.76
MAS	46°41′N, 10°39′E	2300	LARCHES	21	0.465	0.948	254	0.80
MAN	46°44′N, 10°40′E	2250	LARCHES	19	0.519	0.953	182	0.89
KAS	47°21′N, 11°28′E	1900	LARCHES	20	0.388	0.927	247	0.59
KAN	47°21′N, 11°22′E	1700	LARCHES	18	0.539	0.959	208	0.85
TA	46°42′N, 10°20′E	2250	LARCHES	26	0.478	0.96	229	0.74

Table S2. Pearson correlations calculated in CDendro between the tree-ring width series of the five trees selected for wood anatomical analyses and the corresponding site- and species-specific master tree-ring width chronologies. Mean, minimum and maximum *r* values summarize the five individual-to-master correlations calculated for each site and species. Higher *r* values indicate closer agreement with the common growth signal, while minimum and maximum values show the range among selected trees.

SITE	SPECIES	MEAN R	MIN R	MAX R
MAN	PINES	0.59	0.50	0.68
MAN	LARCHES	0.67	0.51	0.77
MAS	PINES	0.59	0.45	0.74
MAS	LARCHES	0.63	0.53	0.76

KAN	PINES	0.60	0.53	0.68
KAN	LARCHES	0.78	0.73	0.85
KAS	PINES	0.61	0.54	0.68
KAS	LARCHES	0.69	0.65	0.76
TA	PINES	0.73	0.68	0.75
TA	LARCHES	0.82	0.78	0.84

Table S3. Statistical data for site chronologies of wood anatomical traits for the study period 1980-2022. RBAR represents the mean inter-series correlation, while EPS (Expressed Population Signal) indicates the robustness of each chronology in representing the population signal.

SITE	SPECIES	TRAIT	N. TREES	RBAR	EPS
MAS	PINES	LWCWT	5	0.231	0.6
KAS	PINES	LWCWT	5	0.324	0.705
KAN	PINES	LWCWT	5	0.185	0.531
MAN	PINES	LWCWT	5	0.216	0.58
TA	PINES	LWCWT	5	0.203	0.559
MAS	LARCHES	LWCWT	5	0.428	0.789
KAS	LARCHES	LWCWT	5	0.414	0.739
KAN	LARCHES	LWCWT	5	0.344	0.724
MAN	LARCHES	LWCWT	5	0.51	0.839
TA	LARCHES	LWCWT	5	0.322	0.704
MAS	PINES	EWLA	5	0.264	0.642
KAS	PINES	EWLA	5	0.355	0.734
KAN	PINES	EWLA	5	0.27	0.649
MAN	PINES	EWLA	5	0.11	0.382
TA	PINES	EWLA	5	0.186	0.533
MAS	LARCHES	EWLA	5	0.217	0.581
KAS	LARCHES	EWLA	5	0.313	0.645
KAN	LARCHES	EWLA	5	0.188	0.536
MAN	LARCHES	EWLA	5	0.334	0.715
TA	LARCHES	EWLA	5	0.343	0.723

Table S4. Collinearity diagnostics among climatic predictors for the March–August window (day of year 60–240; 1980–2022, n = 43 years). Values are Pearson correlations (r) and variance inflation factors (VIFs). T = mean temperature, P = precipitation, RH = relative humidity, and VPD = vapour pressure deficit. Panel A includes T, P and VPD; Panel B additionally includes RH to assess the dependency structure underlying VPD.

A) Diagnostics using the predictors included in the climate-growth analysis

Site	r(VPD, T)	r(VPD, P)	r(T, P)	VIF T	VIF P	VIF VPD
MA	0.79	−0.37	−0.18	2.82	1.20	3.16
KA	0.77	−0.32	−0.25	2.43	1.11	2.55
TA	0.78	−0.35	−0.18	2.66	1.17	2.94

B) Diagnostics including relative humidity

Site	r(VPD, T)	r(VPD, P)	r(VPD, RH)	r(T, RH)	r(T, P)	r(RH, P)	VIF T	VIF P	VIF RH	VIF VPD
MA	0.79	−0.37	−0.77	−0.43	−0.18	0.38	3.60	1.21	3.25	7.43
KA	0.77	−0.32	−0.76	−0.36	−0.25	0.25	3.39	1.12	3.24	6.99
TA	0.78	−0.35	−0.78	−0.43	−0.18	0.31	3.38	1.18	3.23	7.24

Table S5. Results of Kruskal-Wallis and post-hoc Dunn tests comparing tree age among sites for *P. cembra* and *L. decidua*, and Wilcoxon rank-sum test comparing the two species across all sites. The post-hoc comparisons showed that, for *P. cembra*, TA contained significantly older trees than all other sites ($p < 0.05$), while no significant differences were found among the remaining sites. In *L. decidua*, MAN consisted of significantly younger trees compared with all other sites. When pooling all sites, *L. decidua* individuals were significantly older than *P. cembra* (Wilcoxon rank-sum, W = 7068, $p < 0.001$), although this interspecific pattern was not consistent at MAN and TA.

Species	Kruskal–Wallis χ^2 (df)	p-value	Post-hoc (Dunn test, significant comparisons)	Interpretation
P. cembra	17.09 (4)	0.0019	TA > KAN, KAS, MAN, MAS	TA oldest; others similar
L. decidua	18.76 (4)	0.0009	MAN < KAS, MAS, TA;	MAN youngest; KAS & MAS oldest
**Across species**	Wilcoxon rank-sum (W = 7068)	0.0000179	—	*L. decidua* significantly older than *P. cembra* but not at MAN and TA

Table S6. Final tree-level GAMM specifications and model performance for BAI, EWLA and LWCWT. The table reports the response variable, climatic window, structural/ontogenetic controls, temporal smooths, tree-level random effects, AR(1) correction, sample size and model performance. BAI: basal area increment; EWLA: earlywood lumen area; LWCWT: latewood cell wall thickness. The BAI model excludes height because its effect was not significant and performance was unchanged.

**Proxy**	**Response**	**Climate window and climate terms**	**Structural/ontogenetic controls**	**Temporal smooth and random effect**	**AR(1) rho**	**n / trees**	**Deviance explained (%)**	**Adj. R^2**
BAI	log_BAI	May–July (T, P, VPD); Pinus-specific DJF and MAM VPD	s(cambial_age_z, by species, k=10); s(diam_rec_z, by species, k=10)	s(year_c, by site_species, k=40); s(tree_id)	0.539	10,162 / 140	82.8	0.824
EWLA	log_EWLA	May–June (T, P, VPD)	s(cambial_age_z, by species, k=10); s(diam_rec_z, by species, k=10); height_z	s(year_c, by site_species, k=10); s(tree_id)	0.352	2,040 / 50	92.9	0.926
LWCWT	log_LWCWT	July–August (T, P, VPD)	s(cambial_age_z, by = species, k = 10); s(diam_rec_z, by = species, k = 10)	s(year_c, by = site_species, k = 6); s(tree_id)	0.254	2,040 / 50	92.1	0.918

Table S7. Complete formulas of the final tree-level GAMMs. All continuous predictors were standardized before model fitting. In the BAI model, VPD_DJF_pinus_z and VPD_MAM_pinus_z represent standardized winter (December–February) and spring (March–May) VPD predictors defined specifically for *Pinus cembra* and therefore contributing only to observations of this species. The term species:VPD_MJJ_z estimates separate May–July VPD effects for *Larix decidua* and *P. cembra*. All models included AR(1) correction using tree-level AR.start values.

BAI:

log_BAI ~ site_species + s(cambial_age_z, by = species, k = 10) + s(diam_rec_z, by = species, k = 10) + T_MJJ_z + P_MJJ_z + species:VPD_MJJ_z + VPD_DJF_pinus_z + VPD_MAM_pinus_z + s(year_c, by = site_species, k = 40) + s(tree_id, bs = "re")

EWLA:

log_EWLA ~ site_species + s(cambial_age_z, by = species, k = 10) + s(diam_rec_z, by = species, k = 10) + height_z + T_MJ_z + P_MJ_z + species:VPD_MJ_z + s(year_c, by = site_species, k = 10) + s(tree_id, bs = "re")

LWCWT:

log_LWCWT ~ site_species + s(cambial_age_z, by = species, k = 10) + s(diam_rec_z, by = species, k = 10) + T_JA_z + P_JA_z + species:VPD_JA_z + s(year_c, by = site_species, k = 6) + s(tree_id, bs = "re")

Table S8. Candidate model comparison for BAI and EWLA. Models were compared using AIC, ΔAIC, deviance explained and adjusted R^2 to evaluate whether additional structural or climatic terms improved model performance. For BAI, height was not retained despite slightly lower AIC in the height diagnostic model, because its effect was not significant and model performance was unchanged. For EWLA, height was retained because it was significant and biologically relevant.

**Proxy**	**Candidate model**	**AIC**	**ΔAIC**	**Deviance explained (%)**	**Adj. R^2**
BAI	MJJ + Pinus DJF + MAM VPD + height	−404.78	0.00	82.8	0.824
BAI	MJJ + Pinus DJF + MAM VPD	−404.43	0.35	82.8	0.824
BAI	MJJ + Pinus DJF VPD	−401.54	3.25	82.7	0.824
BAI	MJJ + Pinus MAM VPD	−331.04	73.75	82.6	0.823

Proxy	Candidate model	AIC	ΔAIC	Deviance explained (%)	Adj. R^2
BAI	MJJ only	−287.68	117.10	82.6	0.822
EWLA	Age + diameter + height	−3796.09	0.00	92.9	0.926
EWLA	Age + height	−3796.09	0.0003	92.9	0.926
EWLA	Age only	−3795.20	0.89	92.9	0.926
EWLA	Age + diameter	−3795.12	0.97	92.9	0.926
EWLA	Diameter only	−3773.82	22.27	92.7	0.924
EWLA	Height only	−3768.46	27.62	92.6	0.924
EWLA	Diameter + height	−3768.26	27.82	92.6	0.924

Table S9. Collinearity diagnostics for predictors used in the final tree-level GAMMs for BAI, EWLA and LWCWT. The table summarizes the main correlations among climatic and structural predictors, together with VIF/GVIF diagnostics used to assess whether predictors could be retained in the final models. BAI collinearity diagnostics included height in the diagnostic model although height was not retained in the final BAI model.

Proxy	Main climate correlations	Structural correlations	VIF/GVIF summary	Interpretation
BAI	T_MJJ vs VPD_MJJ = 0.750; VPD_MAM vs T_MJJ = 0.581; VPD_MAM vs VPD_DJF = 0.572	age vs diameter = 0.238; age vs height = 0.174; diameter vs height = 0.021	site_species adj. GVIF = 1.11; continuous VIFs = 1.12–2.58	Height included only in diagnostic collinearity model; not retained in final BAI model.
EWLA	T_MJ vs VPD_MJ = 0.670; P_MJ vs VPD_MJ = −0.301; T_MJ vs P_MJ = −0.203	age vs diameter = 0.226; age vs height = −0.093; diameter vs height = −0.125	site_species adj. GVIF = 1.16; continuous VIFs = 1.11–4.11	Highest continuous VIF was height_z = 4.11; retained because significant and biologically relevant.
LWCWT	T_JA vs VPD_JA = 0.677; P_JA vs	Cambial age and reconstructed stem	site_species adj. GVIF = 1.05;	Climate predictors

Proxy	Main climate correlations	Structural correlations	VIF/GVIF summary	Interpretation
	VPD_JA = −0.462; T_JA vs P_JA = −0.428	diameter included as structural/ontogenetic covariates; measured DBH was tested but excluded because it was highly redundant with reconstructed stem diameter.	continuous VIFs = 1.31–2.30;	showed acceptable collinearity. Structural diagnostics indicated redundancy between measured DBH and reconstructed stem diameter, supporting the exclusion of DBH from the final LWCWT model.

Table S10. Concurvity diagnostics for the final tree-level GAMMs. Values summarize the main concurvity patterns among smooth terms, including structural smooths, site × species temporal smooths and the tree-level random effect.

Proxy	Main concurvity pattern	Estimate range	Interpretation
BAI	High global concurvity for species-specific smooths of cambial age and reconstructed diameter; moderate-to-high global concurvity for site-specific temporal smooths	Structural smooths: 0.92–0.98; temporal smooths: 0.88–0.89; tree_id: 0.17	Structural smooths were retained as joint ontogenetic/size-related controls, but not interpreted independently.
EWLA	High global concurvity for species-specific smooths of cambial age and reconstructed diameter; high global concurvity for several site-specific temporal smooths	Structural smooths: 0.97–0.99; temporal smooths: 0.89–0.91; tree_id: 0.56	Strong structural concurvity; individual age and diameter smooths were not interpreted mechanistically.
LWCWT	High concurvity for ontogenetic/size smooths; moderate-to-high concurvity for site-specific temporal smooths	Ontogenetic/size smooths: 0.972–0.993; temporal smooths: 0.893–0.919; tree_id: 0.541	Concurvity mainly reflected the shared developmental and temporal structure captured by cambial age, reconstructed stem diameter and year. These smooths were therefore interpreted jointly as

Proxy	Main concurvity pattern	Estimate range	Interpretation
			structural controls rather than as independent effects; climate fixed effects remained interpretable after accounting for this structure.

Table S11. Sensitivity of fixed effects to alternative structural specifications in the BAI and EWLA GAMMs.

Proxy	Term	Final model	Without cambial age	Without reconstructed diameter	Interpretation
BAI	T_MJJ_z	0.0340***	0.0339***	0.0326***	Stable positive effect
BAI	P_MJJ_z	−0.0072**	−0.0074**	−0.0075**	Stable negative effect
BAI	VPD_DJF_pinus_z	−0.0368***	−0.0375***	−0.0367***	Stable negative effect
BAI	VPD_MAM_pinus_z	−0.0091*	−0.0088*	−0.0087*	Stable weak negative effect
BAI	Larix:VPD_MJJ_z	0.0552***	0.0560***	0.0577***	Stable positive effect
BAI	Pinus:VPD_MJJ_z	0.0164***	0.0162**	0.0177***	Stable positive effect
EWLA	height_z	0.0789*	0.0699*	0.0789*	Stable positive effect
EWLA	T_MJ_z	0.0004 ns	0.0009 ns	0.0004 ns	Not significant
EWLA	P_MJ_z	−0.0009 ns	−0.0011 ns	−0.0009 ns	Not significant
EWLA	Larix:VPD_MJ_z	0.0080*	0.0077*	0.0080*	Stable weak positive effect
EWLA	Pinus:VPD_MJ_z	0.0006 ns	0.0003 ns	0.0006 ns	Not significant

Table S12. Standardized fixed effects retained for interpretation in the final GAMMs. Estimates are reported on the log scale and expressed as percent change per +1 SD increase in the predictor. Intercepts and site × species contrasts are omitted for brevity. Significance codes: *** p < 0.001; ** p < 0.01; * p < 0.05; ns = not significant. SE= standard error; *t* = test statistic;

Proxy	Term	Estimate	SE	t	p	Percent change per +1 SD (%)	Sig.
BAI	speciesLarix_decidua:VPD_MJJ_z	0.0552	0.00464	11.91	1.75e−32	+5.68	***
BAI	VPD_DJF_pinus_z	−0.0368	0.00420	−8.76	2.21e−18	−3.61	***
BAI	T_MJJ_z	0.0340	0.00449	7.58	3.70e−14	+3.46	***
BAI	speciesPinus_cembra:VPD_MJJ_z	0.0164	0.00496	3.31	9.53e−04	+1.65	***
BAI	VPD_MAM_pinus_z	−0.00909	0.00420	−2.16	0.0306	−0.91	*
BAI	P_MJJ_z	−0.00716	0.00250	−2.87	0.00413	−0.71	**
EWLA	height_z	0.0789	0.0328	2.41	0.0162	+8.21	*
EWLA	speciesLarix_decidua:VPD_MJ_z	0.00804	0.00345	2.33	0.0200	+0.81	*
EWLA	P_MJ_z	−0.000922	0.00235	−0.39	0.695	−0.09	ns
EWLA	speciesPinus_cembra:VPD_MJ_z	0.000573	0.00352	0.16	0.871	+0.06	ns
EWLA	T_MJ_z	0.000444	0.00318	0.14	0.889	+0.04	ns
LWCWT	T_JA_z	0.0173	0.00263	6.58	6.06e−11	+1.74	***
LWCWT	P_JA_z	0.00467	0.00140	3.34	8.46e−04	+0.47	***
LWCWT	speciesLarix_decidua:VPD_JA_z	0.00328	0.00273	1.20	0.230	+0.33	ns
LWCWT	speciesPinus_cembra:VPD_JA_z	−0.00253	0.00279	−0.91	0.364	−0.25	ns

Table S13. Approximate significance of smooth terms in the final GAMMs. The table reports estimated degrees of freedom (edf), reference degrees of freedom (Ref.df), F statistics and p values for structural, temporal and tree-level random-effect smooths. Small edf values close to zero indicate that the smoothing penalty effectively removed the term. The tree_id smooth is the tree-level random effect.

Proxy	Smooth term	edf	Ref.df	F	p	Sig.
BAI	s(cambial_age_z):Larix_decidua	6.09	9	1162.79	1.33e−04	***
BAI	s(cambial_age_z):Pinus_cembra	6.22	9	2179.52	<2e−16	***
BAI	s(diam_bai_z):Larix_decidua	6.08	9	8668.99	<2e−16	***

Proxy	Smooth term	edf	Ref.df	F	p	Sig.
BAI	s(diam_bai_z):Pinus_cembra	4.60	9	4759.89	<2e−16	***
BAI	s(year_c):KAN.Larix_decidua	8.55	9	16.79	<2e−16	***
BAI	s(year_c):KAS.Larix_decidua	7.06	9	10.05	7.28e−06	***
BAI	s(year_c):MAN.Larix_decidua	8.53	9	22.45	<2e−16	***
BAI	s(year_c):MAS.Larix_decidua	8.62	9	16.63	<2e−16	***
BAI	s(year_c):TA.Larix_decidua	8.56	9	33.76	<2e−16	***
BAI	s(year_c):KAN.Pinus_cembra	3.05	9	2.01	0.00446	**
BAI	s(year_c):KAS.Pinus_cembra	2.89	9	4.50	2.29e−06	***
BAI	s(year_c):MAN.Pinus_cembra	3.69	9	2.80	1.87e−04	***
BAI	s(year_c):MAS.Pinus_cembra	6.84	9	9.75	<2e−16	***
BAI	s(year_c):TA.Pinus_cembra	0.00	9	0.00	0.893	ns
BAI	s(tree_id)	121.75	135	25.28	<2e−16	***
EWLA	s(cambial_age_z):Larix_decidua	0.00	9	0.00	0.782	ns
EWLA	s(cambial_age_z):Pinus_cembra	4.64	9	273.50	5.62e−05	***
EWLA	s(diam_rec_z):Larix_decidua	0.00	9	0.00	0.815	ns
EWLA	s(diam_rec_z):Pinus_cembra	0.00	9	0.00	0.704	ns
EWLA	s(year_c):KAN.Larix_decidua	0.90	9	1.01	0.00151	**
EWLA	s(year_c):KAS.Larix_decidua	6.37	9	6.07	2.74e−06	***
EWLA	s(year_c):MAN.Larix_decidua	1.90	9	1.56	4.43e−04	***
EWLA	s(year_c):MAS.Larix_decidua	1.58	9	2.78	7.13e−07	***
EWLA	s(year_c):TA.Larix_decidua	0.00	9	0.00	0.792	ns
EWLA	s(year_c):KAN.Pinus_cembra	0.00	9	0.00	0.313	ns
EWLA	s(year_c):KAS.Pinus_cembra	2.07	9	2.80	1.01e−05	***
EWLA	s(year_c):MAN.Pinus_cembra	2.06	9	1.38	0.00242	**
EWLA	s(year_c):MAS.Pinus_cembra	0.57	9	0.30	0.133	ns
EWLA	s(year_c):TA.Pinus_cembra	0.54	9	0.09	0.234	ns
EWLA	s(tree_id)	35.27	38	23.14	<2e−16	***
LWCWT	s(cambial_age_z):Larix_decidua	4.43	9	95.03	0.117	ns
LWCWT	s(cambial_age_z):Pinus_cembra	5.99	9	178.89	0.000195	***
LWCWT	s(diam_rec_z):Larix_decidua	0.40	9	11.86	0.188	ns

Proxy	Smooth term	edf	Ref.df	F	p	Sig.
LWCWT	s(diam_rec_z):Pinus_cembra	2.31	8	306.64	0.0235	*
LWCWT	s(year_c):KAN.Larix_decidua	0.70	5	0.61	0.0612	.
LWCWT	s(year_c):KAS.Larix_decidua	0.88	5	2.91	0.00236	**
LWCWT	s(year_c):MAN.Larix_decidua	0.00	5	0.00	0.338	ns
LWCWT	s(year_c):MAS.Larix_decidua	3.83	5	6.11	6.43e−05	***
LWCWT	s(year_c):TA.Larix_decidua	0.78	5	0.92	0.0242	*
LWCWT	s(year_c):KAN.Pinus_cembra	2.16	5	6.50	7.32e−06	***
LWCWT	s(year_c):KAS.Pinus_cembra	0.00	5	0.00	0.479	ns
LWCWT	s(year_c):MAN.Pinus_cembra	0.52	5	0.16	0.220	ns
LWCWT	s(year_c):MAS.Pinus_cembra	2.81	5	15.10	<2e−16	***
LWCWT	s(year_c):TA.Pinus_cembra	2.56	5	5.17	1.98e−05	***
LWCWT	s(tree_id)	34.39	38	24.08	<2e−16	***

Table S14. Relative importance of components in the final BAI model based on component-removal comparisons. Each reduced model was obtained by removing one model component at a time from the final BAI model. Changes in AIC, deviance explained and adjusted R^2 indicate the loss of explanatory performance associated with each component. Component importance was assessed by comparing the final BAI model with reduced models in which one component was removed at a time.

Removed component	AIC	ΔAIC from full	Deviance explained	Loss in deviance explained (%)	Adj. R^2	Loss in adj. R^2 (%)
Residual temporal smooths	335	740	0.791	3.67	0.788	3.62
All climate	396	800	0.818	0.92	0.814	0.93
MJJ climate	299	703	0.820	0.74	0.816	0.75
Reconstructed diameter	−244	161	0.822	0.60	0.818	0.59
Cambial age	−297	107	0.822	0.57	0.818	0.57
Pinus DJF + MAM VPD	−288	117	0.826	0.19	0.822	0.18

Table S15. Fitted component ranges for the final BAI, LWCWT, EWLA model on the response scale. Values represent the minimum-to-maximum amplitude of each fitted model component across observations. Large ranges for reconstructed diameter reflect the multiplicative transformation from the log scale. Values represent fitted component ranges on the response scale and describe the amplitude of each model component across observations. They are not variance explained. Large structural ranges, especially for reconstructed diameter, reflect the multiplicative transformation from the log scale and the intrinsic geometric dependence of BAI on stem size; therefore, cambial age and reconstructed diameter were interpreted as ontogenetic and size-related controls rather than independent mechanistic drivers. Because values were back-transformed from the log scale, they can exceed 100%.

BAI

**Component**	**Larix decidua range (%)**	**Pinus cembra range (%)**	**Interpretation**
Climate all	45.7–46.2	26.5–32.7	Realized fitted climate contribution
Cambial age	76.8–94.2	135–208	Ontogenetic control
Reconstructed diameter	187–6806	154–573	Size/geometric control
Residual temporal smooth	53.4–171	~0–78.3	Unexplained site-specific temporal dynamics
Tree random effect	66.2–350	127–297	Individual-tree variability
Site × species fixed effect	10.2%	72.2%	Between-site baseline differences

LWCWT

**Component**	**Larix decidua range (%)**	**Pinus cembra range (%)**	**Interpretation**
Climate all	7.2–7.6	5.8–6.1	Realized fitted climate contribution
Reconstructed diameter	0.96–2.91	0.78–1.63	Size/geometric control
Residual temporal smooth	~0–6.81	~0–8.23	Unexplained site-specific temporal dynamics

Component	Larix decidua range (%)	Pinus cembra range (%)	Interpretation
Tree random effect	8.0–22.3	10.3–41.0	Individual-tree variability
Site × species fixed effect	22.8	14.5	Between-site baseline differences

EWLA

Component	Larix decidua range (%)	Pinus cembra range (%)	Interpretation
Climate all	0.96–2.91	0.78–1.63	Realized fitted climate contribution
Reconstructed diameter	~0–~0	~0–~0	Size/geometric control
Residual temporal smooth	~0–25.5	~0–15.3	Unexplained site-specific temporal dynamics
Tree random effect	13.1–39.2	17.1–45.5	Individual-tree variability
Site × species fixed effect	35.1	18.2	Between-site baseline differences
Cambial age	~0–~0	3.28–46.4	Ontogenetic control
Tree height	8.37-18.7	4.48-13.6	Height-related structural/path-length control

Table S16. Residual temporal smooth ranges for EWLA and LWCWT. Smooth ranges are expressed as percent change across the fitted site × species temporal smooths and describe residual temporal structure after accounting for structural predictors, climate, site/species differences, tree identity and AR(1) autocorrelation. Smooth ranges are expressed as percent change across the fitted temporal smooth. These values describe residual temporal structure after accounting for structural predictors, climate, site/species differences, tree identity and AR(1) autocorrelation.

Proxy	Site × species	Residual temporal smooth	Smooth range (%)
EWLA	KAN.Larix_decidua	Significant	9.73
EWLA	KAS.Larix_decidua	Significant	25.5
EWLA	MAN.Larix_decidua	Significant	11.5
EWLA	MAS.Larix_decidua	Significant	16.8
EWLA	TA.Larix_decidua	Not significant	~0.0
EWLA	KAN.Pinus_cembra	Not significant	~0.0
EWLA	KAS.Pinus_cembra	Significant	15.3
EWLA	MAN.Pinus_cembra	Significant	12.7
EWLA	MAS.Pinus_cembra	Not significant	4.93
EWLA	TA.Pinus_cembra	Not significant	1.72
LWCWT	KAN.Larix_decidua	Not significant	2.72
LWCWT	KAS.Larix_decidua	Significant	6.20
LWCWT	MAN.Larix_decidua	Not significant	0.0
LWCWT	MAS.Larix_decidua	Significant	9.8
LWCWT	TA.Larix_decidua	Significant	3.5
LWCWT	KAN.Pinus_cembra	Significant	9.2
LWCWT	KAS.Pinus_cembra	Not significant	0.0
LWCWT	MAN.Pinus_cembra	Not significant	0.9
LWCWT	MAS.Pinus_cembra	Significant	13.6
LWCWT	TA.Pinus_cembra	Significant	9.61

Table S17. Linear mixed-effects model structure used to test absolute species differences in TRW, EWLA and LWCWT. The main model was fitted to annual site-level chronology values and included species, proxy, their interaction and region as fixed effects, with site nested within site pair as a random effect, continuous AR(1) temporal autocorrelation within each site × proxy series and proxy-specific residual variances. The slope/aspect sensitivity model excluded S-charl because slope aspect was unavailable.

Item	Main model	Slope/aspect sensitivity model
Response	Annual site-level chronology values	Annual site-level chronology values
Fixed effects	species * proxy + region	species * proxy + region + slope aspect
Random effects	site nested within site pair	site nested within site pair
Temporal autocorrelation	continuous AR(1) within site x proxy series	continuous AR(1) within site x proxy series
Heteroscedasticity	proxy-specific residual variance	proxy-specific residual variance
Observations	1590	1272
Site-level series	30	24
AR(1) phi	0.753	0.716
AIC	4134.66	3300.58

Table S18. ANOVA of the main mixed-effects model testing absolute species differences among proxies. The significant species × proxy interaction indicates that interspecific differences depended on the growth proxy. numDF, numerator degrees of freedom; denDF, denominator degrees of freedom; F, F-test statistic; p, probability value associated with the F test.

Effect	numDF	denDF	F	p
species	1	4	8.916	0.0405
proxy	2	1576	5790.582	<0.0001
region	2	2	0.085	0.9217
species x proxy	2	1576	439.329	<0.0001

Table S19. Estimated marginal means (EMMs) and species contrasts from the main mixed-effects model. EMMs are averaged over regions. Contrasts are calculated as *P. cembra* – *L. decidua*; negative contrasts indicate higher values in *L. decidua*. SE, standard error of the contrast; df, degrees of freedom; Holm *p*, *p*-value adjusted for multiple comparisons using the Holm method. ns = not significant; *** p < 0.001.

Proxy	P. cembra EMM	L. decidua EMM	Contrast (P. cembra - L. decidua)	SE	df	Holm p	Significance
TRW	0.935	0.809	+0.127	0.082	4	0.1975	ns
EWLA	599.3	1161.6	-562.3	24.05	4	5.94e-05	***
LWCWT	3.790	5.376	-1.586	0.109	4	2.56e-04	***

Table S20. Slope/aspect sensitivity analysis for absolute species differences after excluding S-charl. Contrasts are calculated as *P. cembra – L. decidua*. The main inference was unchanged after including slope aspect, with no significant species difference in TRW but significantly higher EWLA and LWCWT in *L. decidua*.

Proxy / effect	Estimate or F	SE / df	Holm p / p	Interpretation
Slope aspect	F = 2.642	1, 1	0.3511	No significant slope-aspect effect
species x proxy	F = 442.018	2, 1260	<0.0001	Species differences remained proxy-dependent
TRW contrast	+0.162	SE = 0.078	0.1300	No significant species difference
EWLA contrast	-558.7	SE = 24.08	5.26e-04	Higher EWLA in L. decidua
LWCWT contrast	-1.478	SE = 0.101	0.00138	Higher LWCWT in L. decidua

Table S21. Empirical climatic envelopes for temperature, precipitation and vapour pressure deficit (VPD) during negative pointer years of tree-ring width (TRW) and earlywood lumen area (EWLA) for each site and species over 1980–2022. Climatic variables were calculated over May–July for TRW (MJJ) and May–June for EWLA (MJ). The column *n* indicates the number of negative pointer years for each proxy–site–species combination. Values represent the interquartile range (Q25–Q75) of observed climatic conditions and are intended as descriptive empirical benchmarks rather than physiological thresholds.

Site	Species	n	Temperature Q25–Q75 (°C)	Precipitation Q25–Q75 (mm)	VPD Q25–Q75 (kPa)
**TRW (MJJ)**					
MAS	*L. decidua*	3	7.78–8.05	315.25–346.02	0.27–0.29
MAS	*P. cembra*	2	7.36–7.74	336.5–353.49	0.29–0.33
MAN	*L. decidua*	1	7.54–7.54	294.16–294.16	0.27–0.27
MAN	*P. cembra*	1	7.8–7.8	289.88–289.88	0.37–0.37
TA	*L. decidua*	4	5.96–7.06	255.8–366.97	0.23–0.25
TA	*P. cembra*	3	6.5–7	356.84–385.46	0.25–0.29
KAS	*L. decidua*	2	8.09–8.47	464.59–493.14	0.28–0.29
KAS	*P. cembra*	2	8.99–9.38	425.27–456.85	0.32–0.33
KAN	*L. decidua*	4	8.3–8.75	429.53–464.59	0.29–0.33
KAN	*P. cembra*	2	9.62–9.71	443.31–510.95	0.34–0.34
**EWLA (MJ)**					
MAS	*L. decidua*	3	5.81–6.44	227.33–249.75	0.23–0.24
MAS	*P. cembra*	4	5.75–7.1	181.5–210.36	0.27–0.31
MAN	*L. decidua*	2	5.83–6.51	161.22–195.65	0.24–0.25
MAN	*P. cembra*	2	5.85–6.52	163.26–201.77	0.25–0.25
TA	*L. decidua*	3	5.33–5.72	262.28–281.25	0.21–0.24
TA	*P. cembra*	3	4.49–5.17	186.3–217.49	0.22–0.24
KAS	*L. decidua*	3	7.38–8.38	281.87–301.73	0.31–0.35
KAS	*P. cembra*	3	7.77–9.01	248.9–277.73	0.28–0.31
KAN	*L. decidua*	4	6.95–9.07	265.77–290.91	0.29–0.34
KAN	*P. cembra*	2	7.66–8.51	265.46–275.8	0.33–0.36
